# Supplementary material for: SUMOylation of AnxA6 facilitates EGFR-PKCα complex formation to suppress epithelial cancer growth
Source: Cell Commun Signal. 2023 Aug 1;21:189. doi: 10.1186/s12964-023-01217-x (PMC10391975; doi:10.1186/s12964-023-01217-x)

Figure 1. A

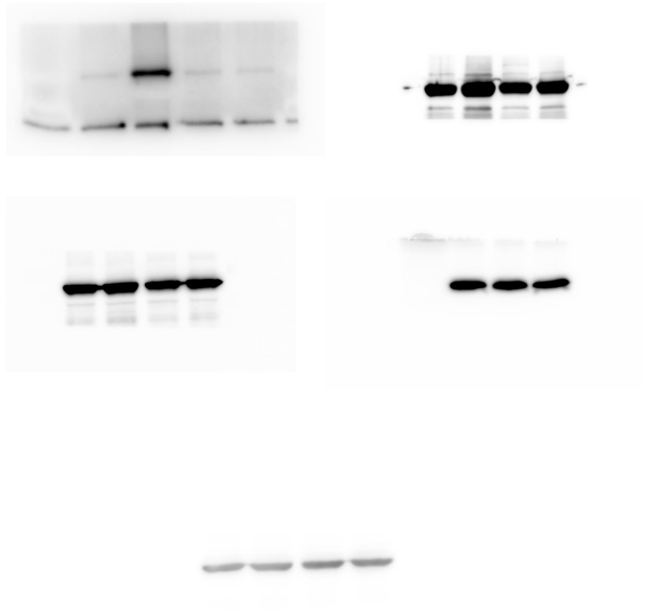

Figure 1. B

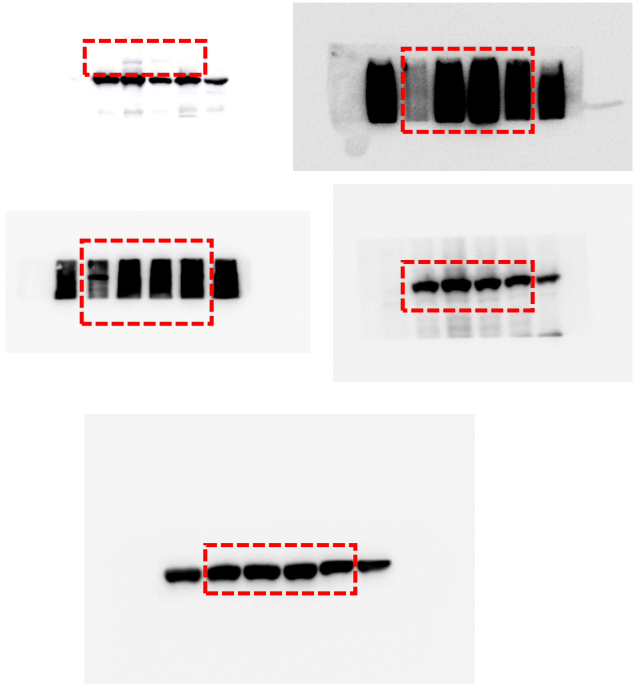

Figure 1. C

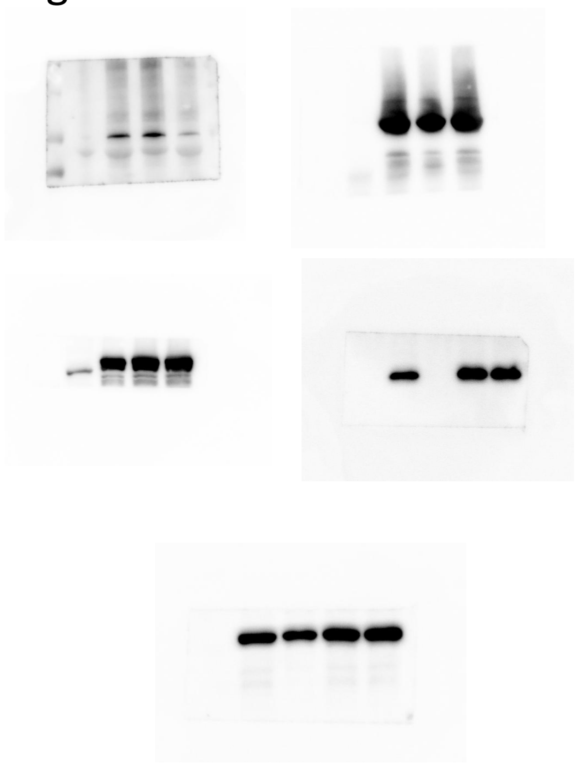

Figure 1. D

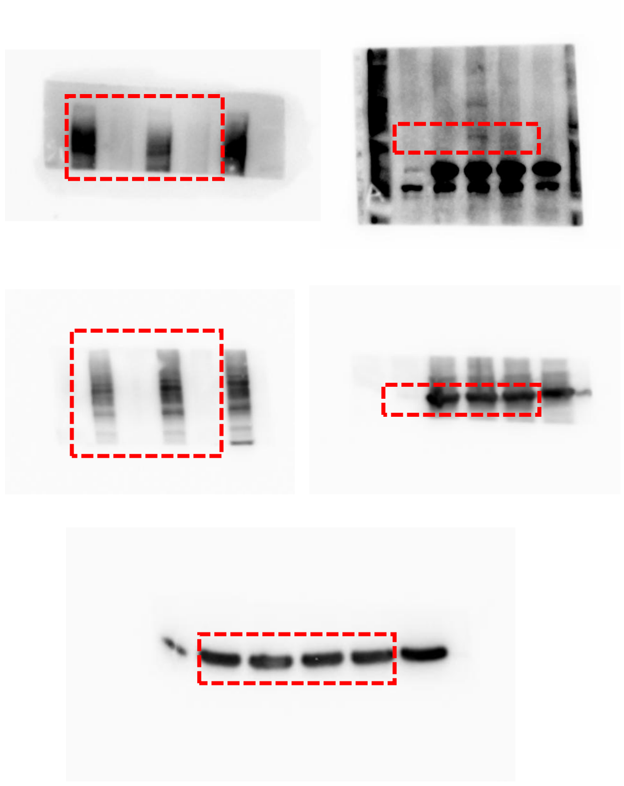

Figure 2. A

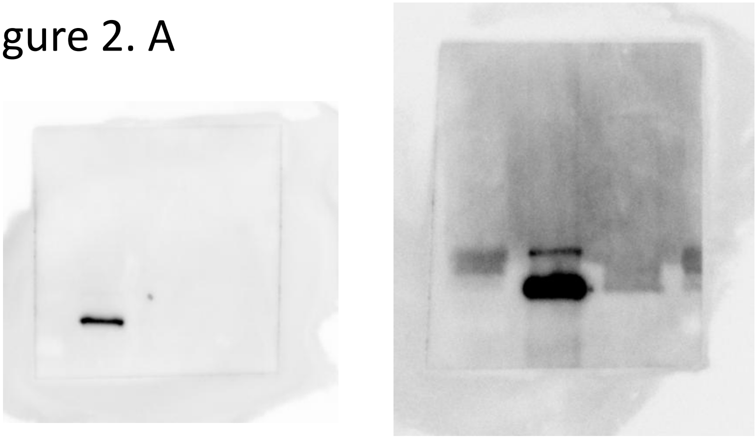

Figure 2. E

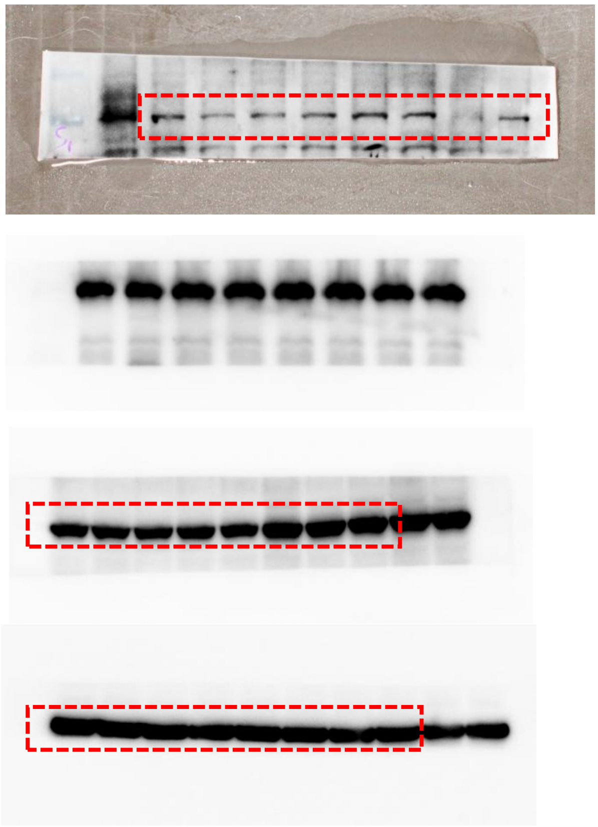

Figure 2. F

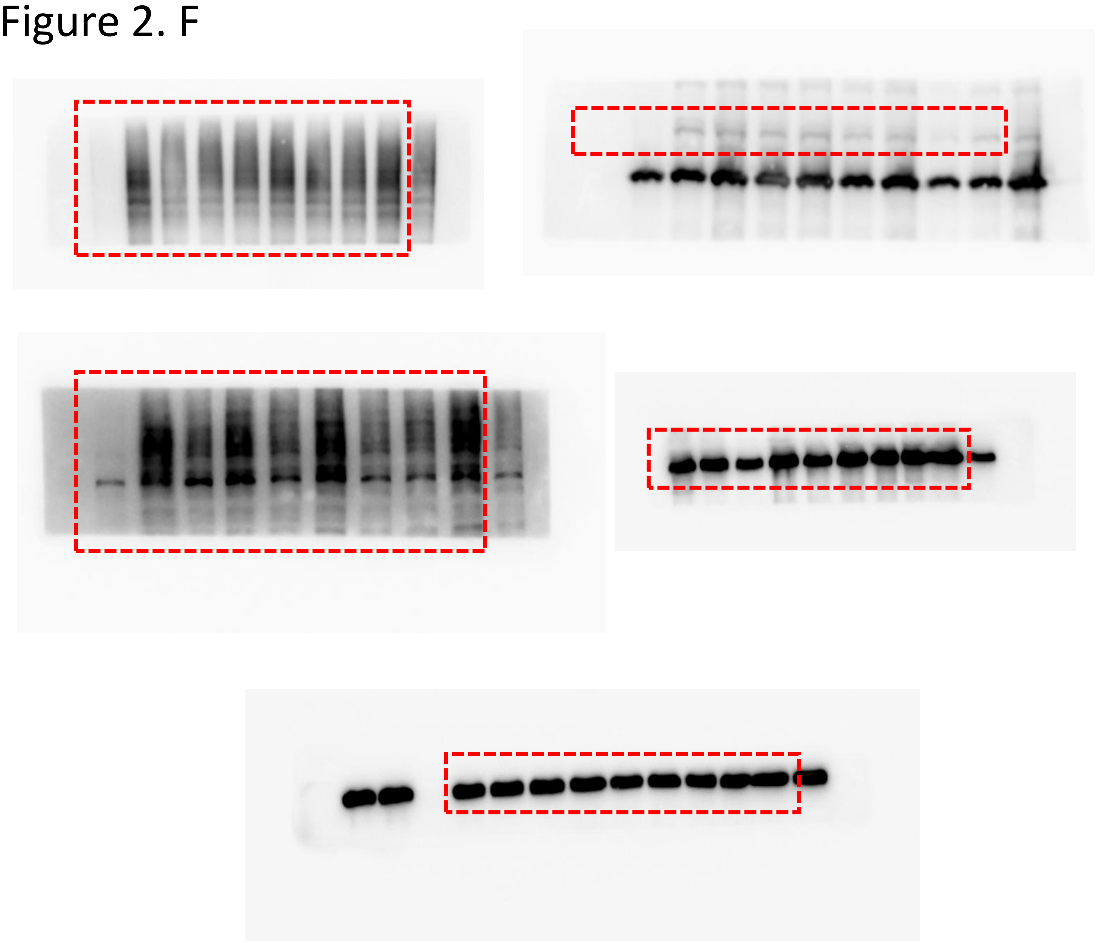

Figure 3. A

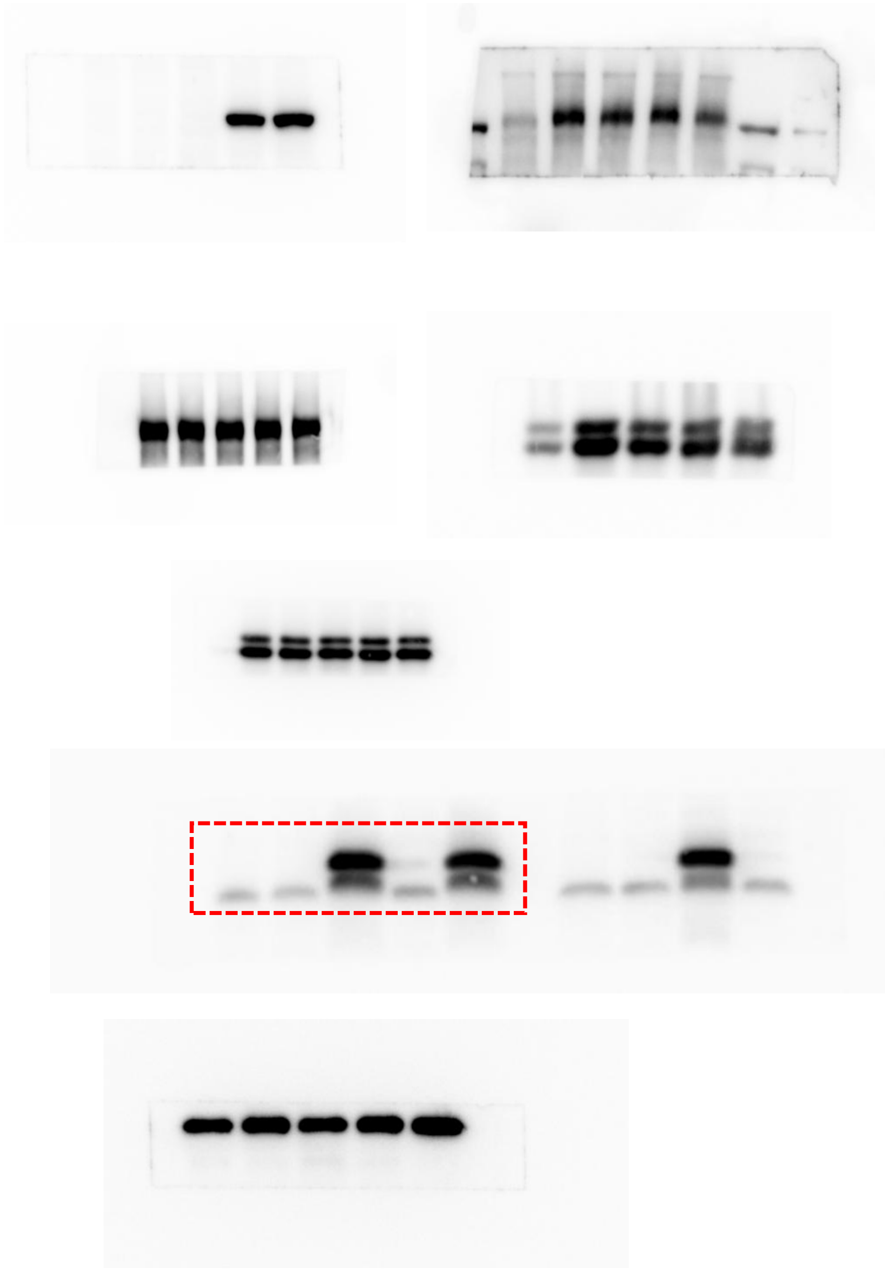

Figure 3. B

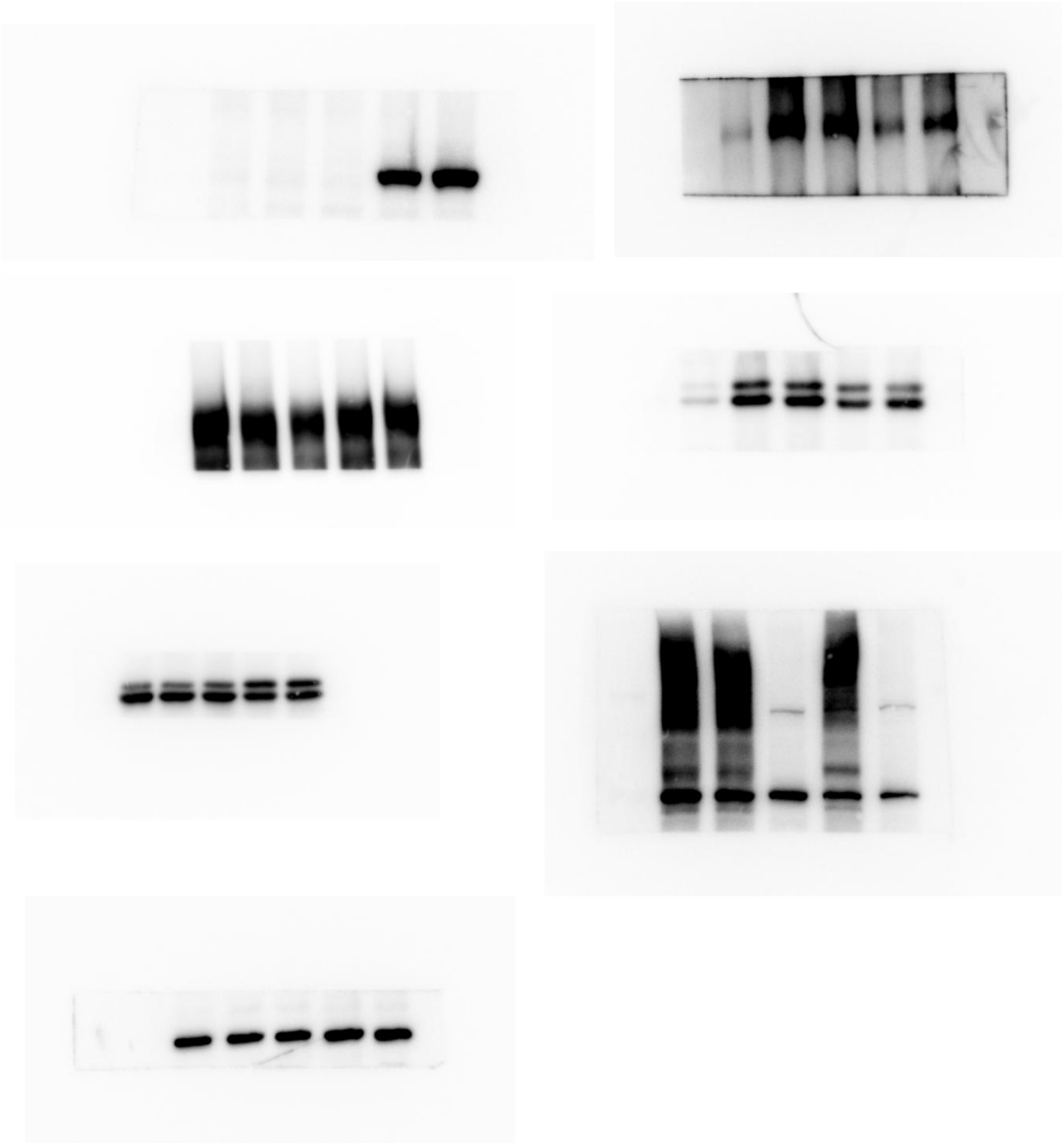

Figure 3. C

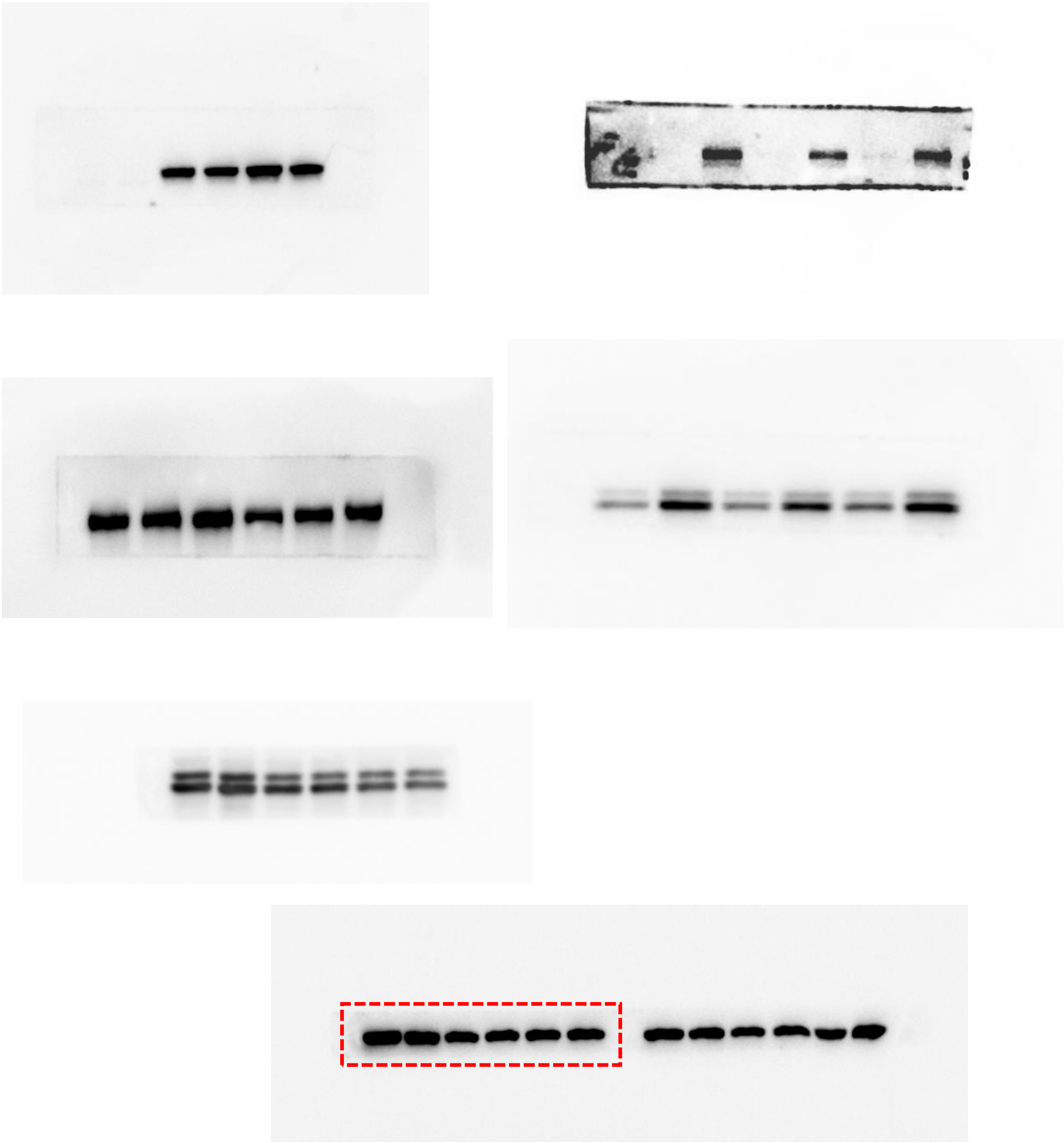

Figure 3. D

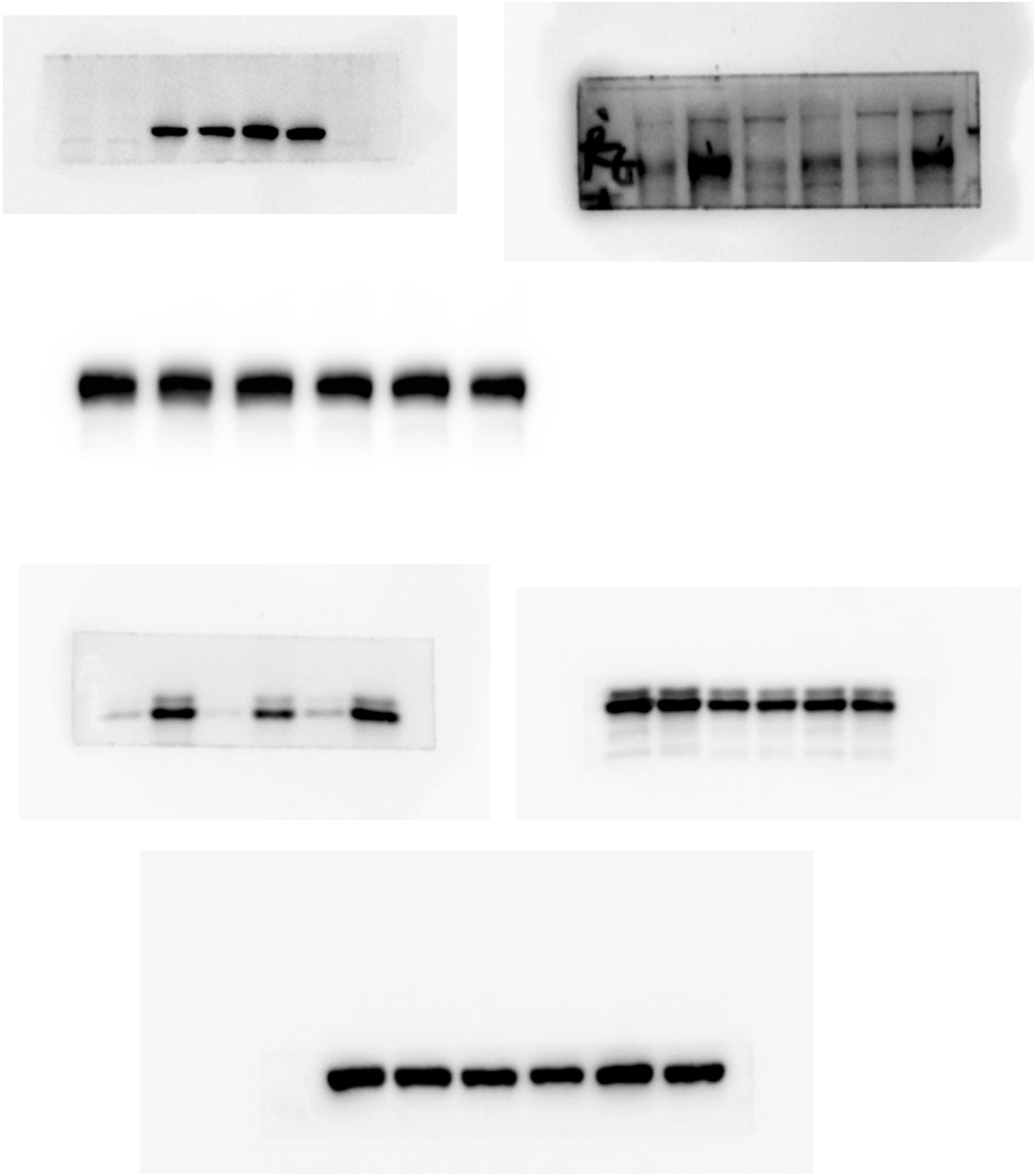

Figure 3. E

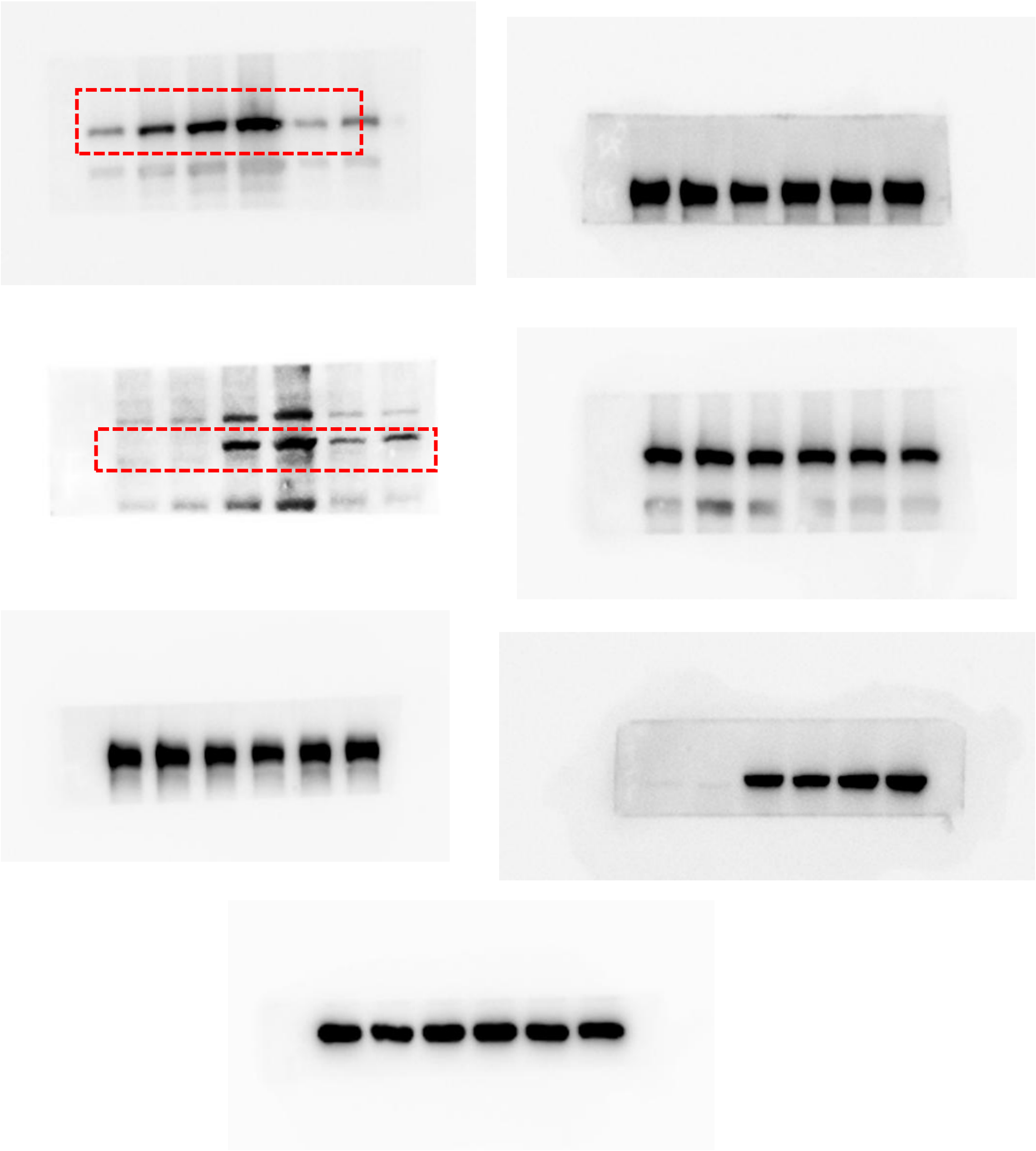

Figure 3. F

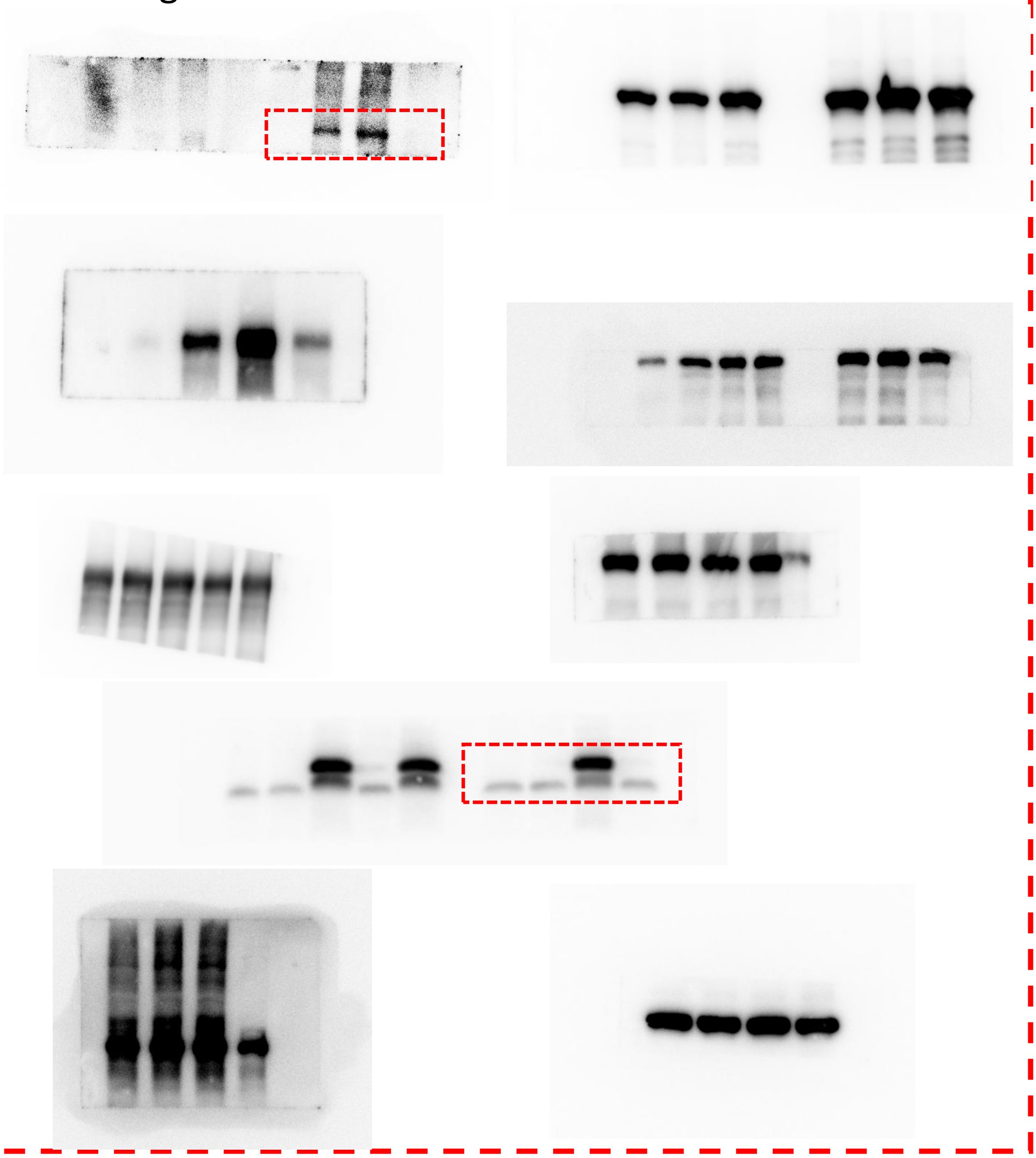

Figure 3. G

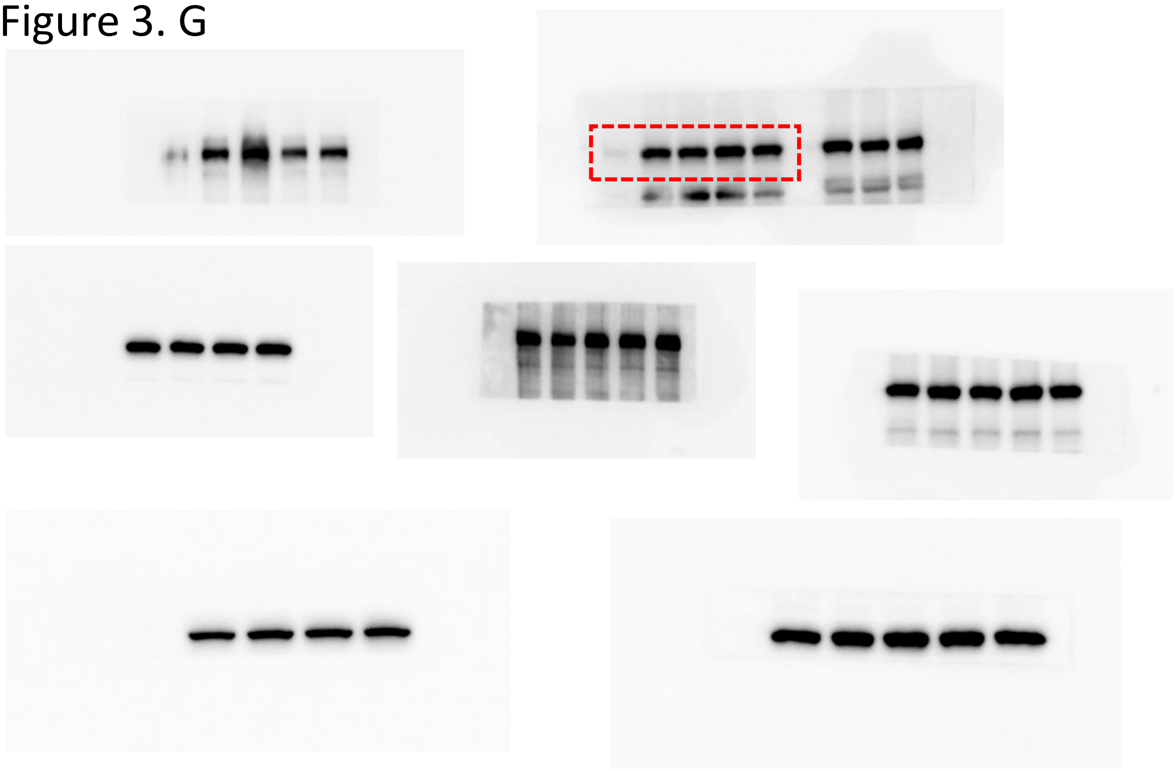

Figure 4A

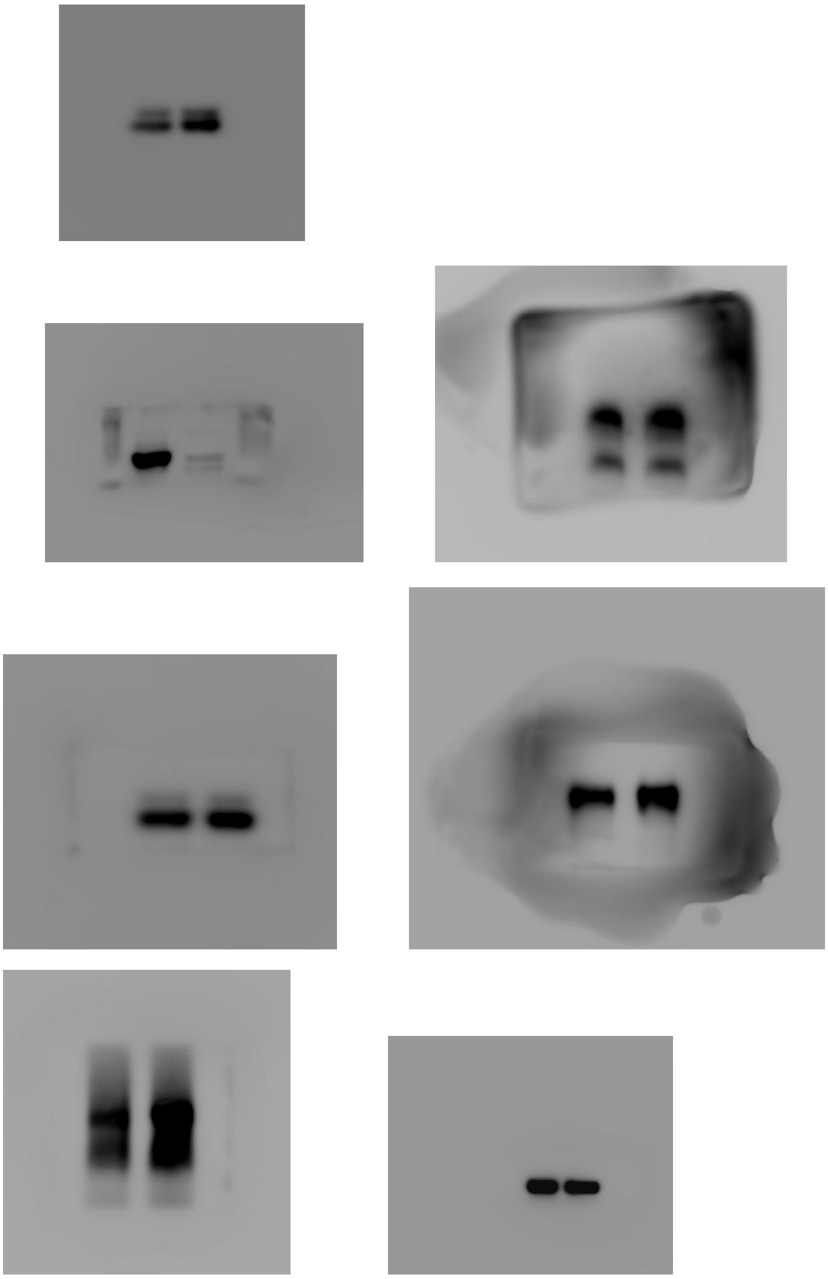

Figure 4B

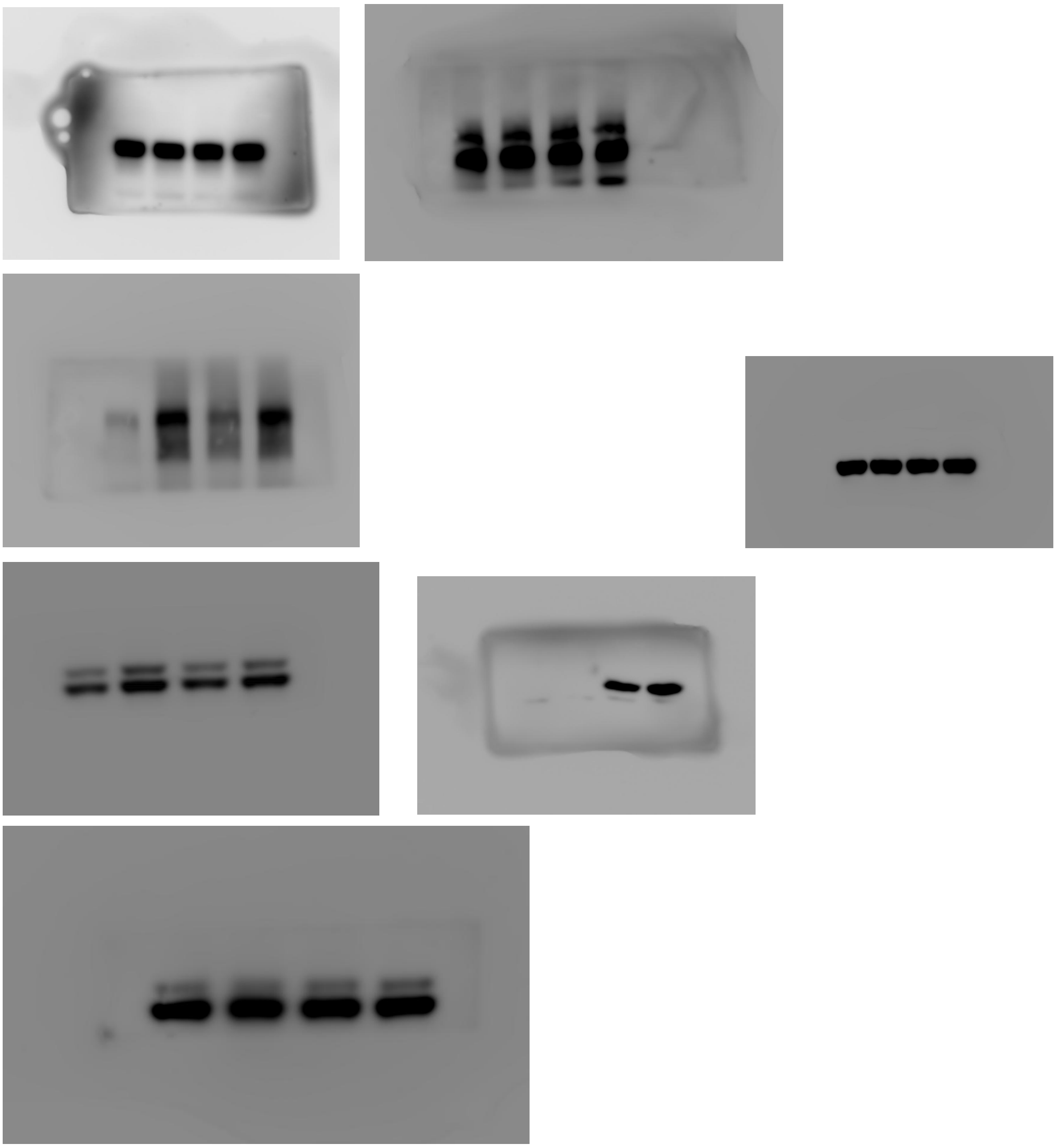

Figure 4C

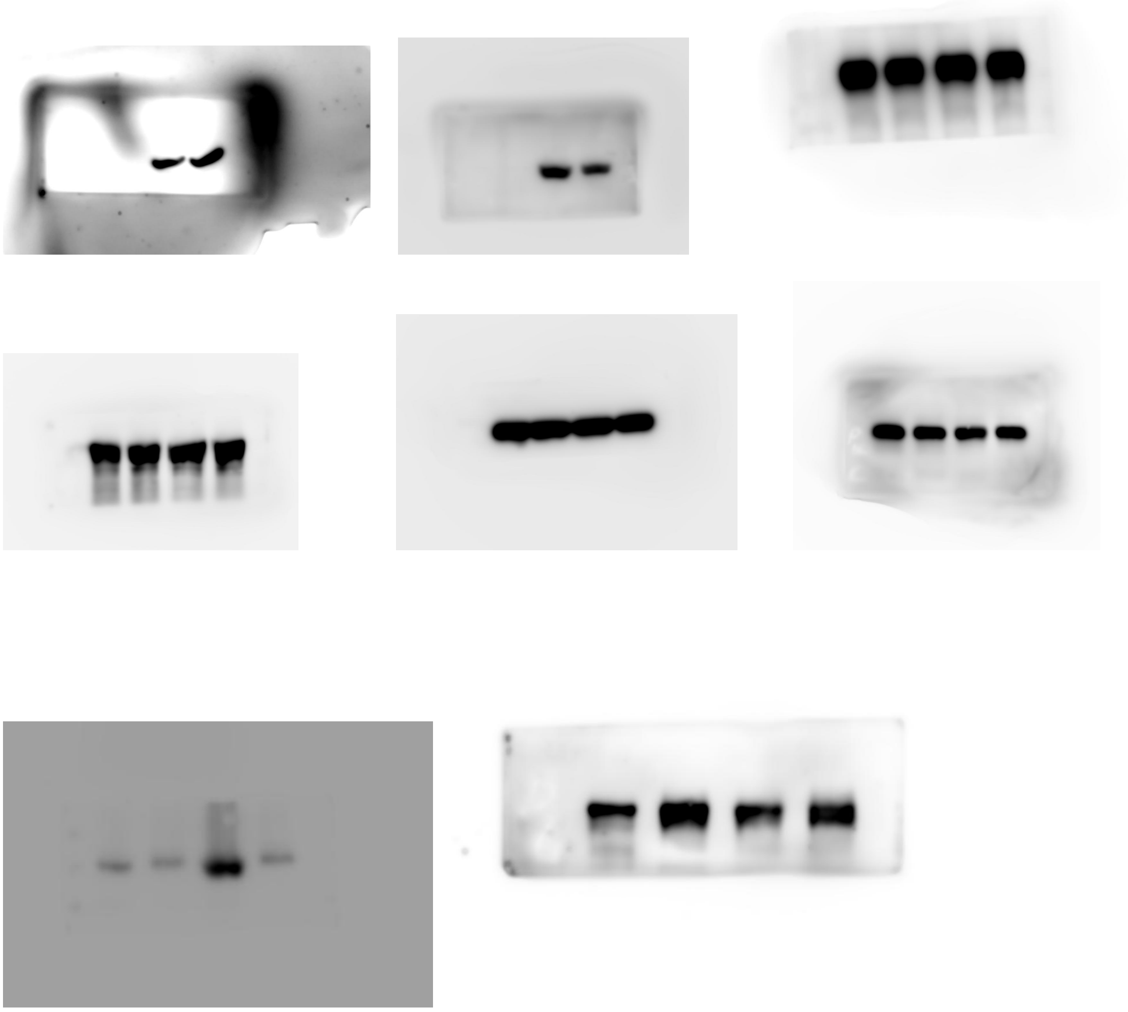

Figure 4D

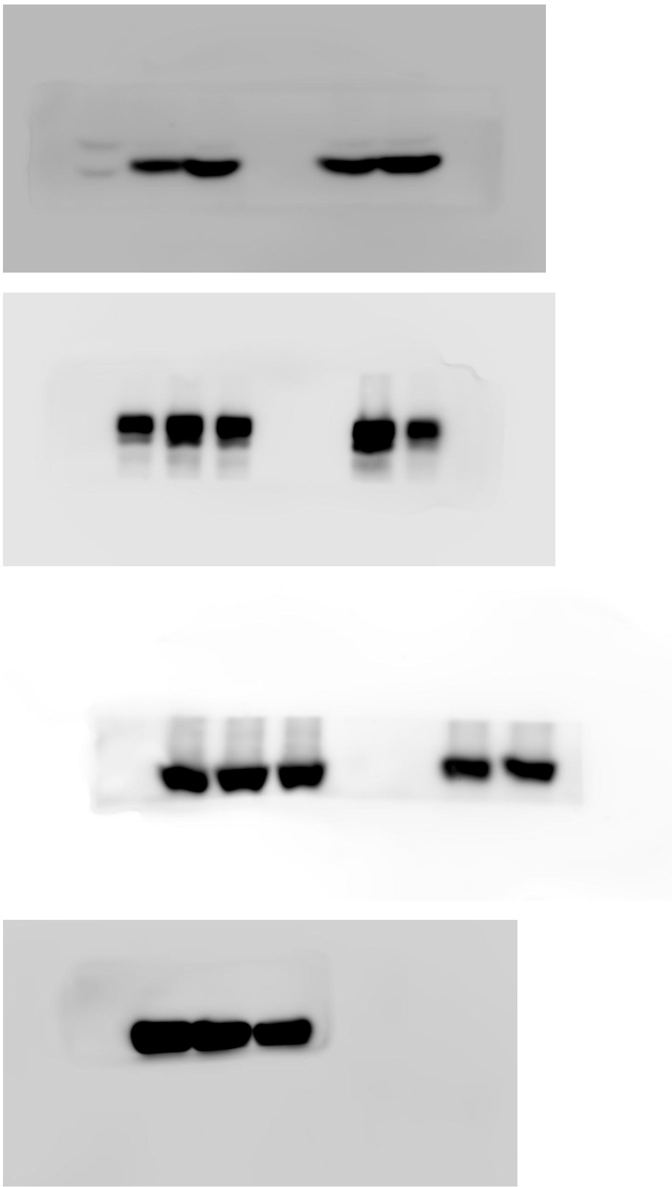

Figure 5. A

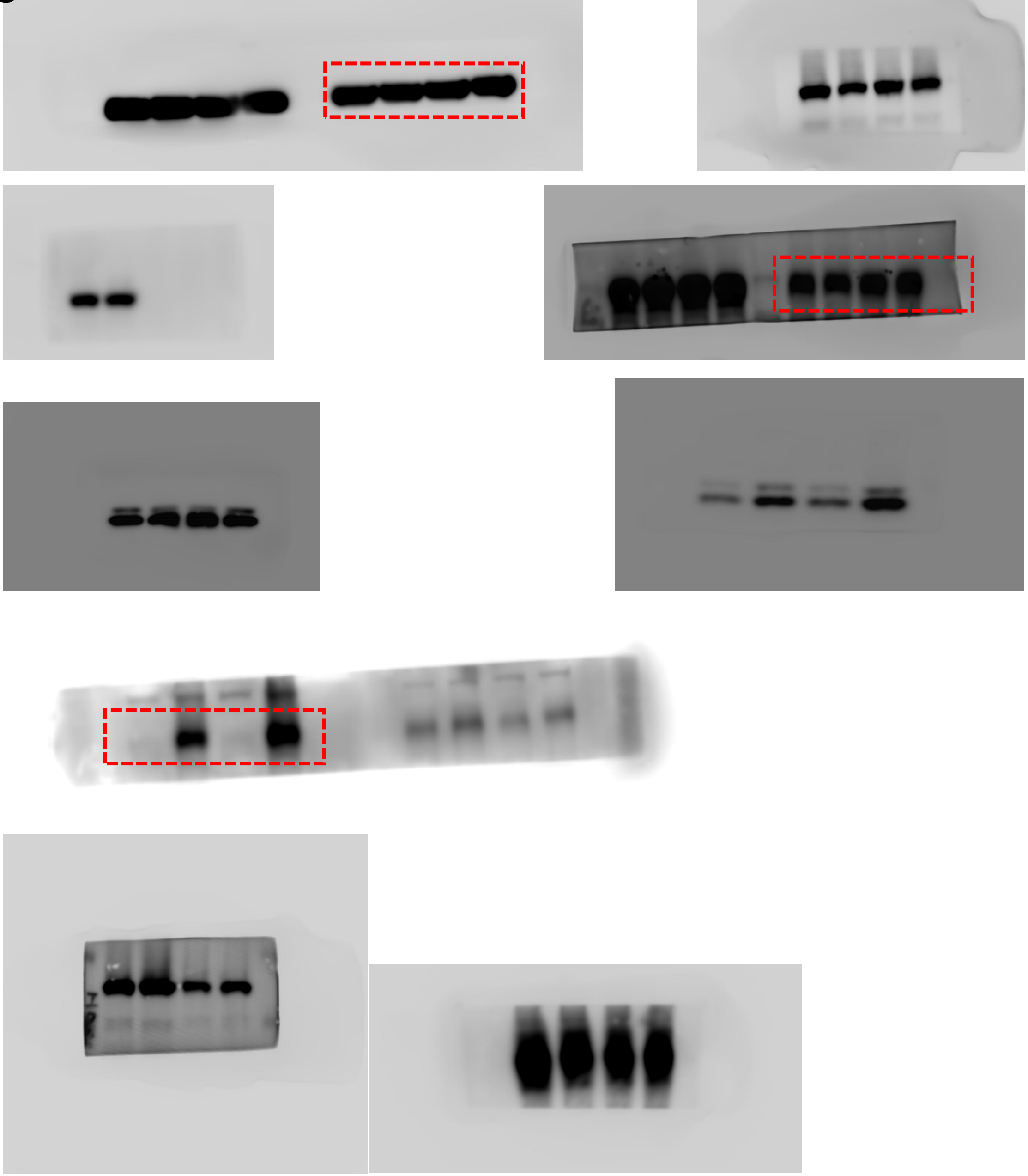

Figure 5. E

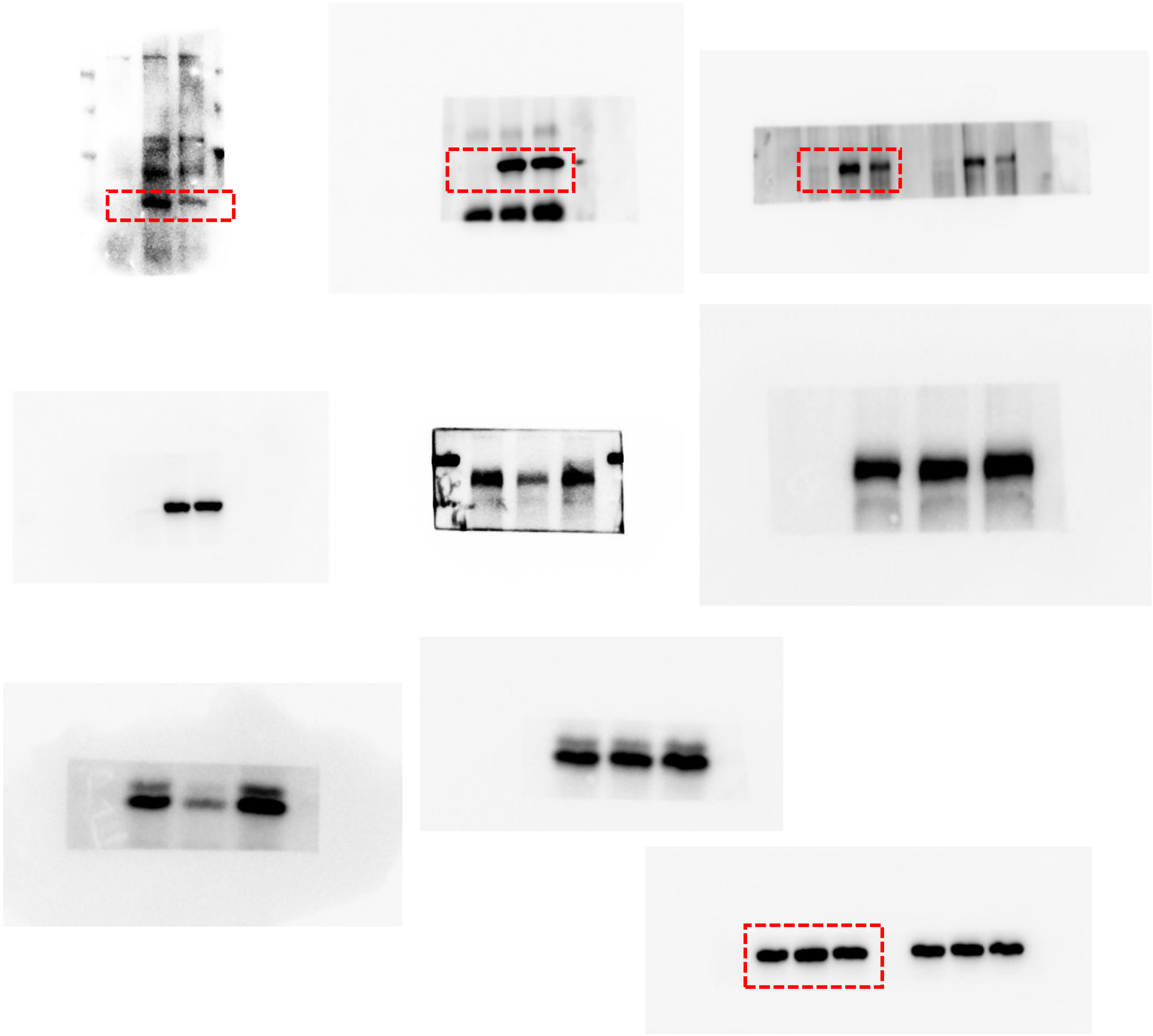

Figure 5. I

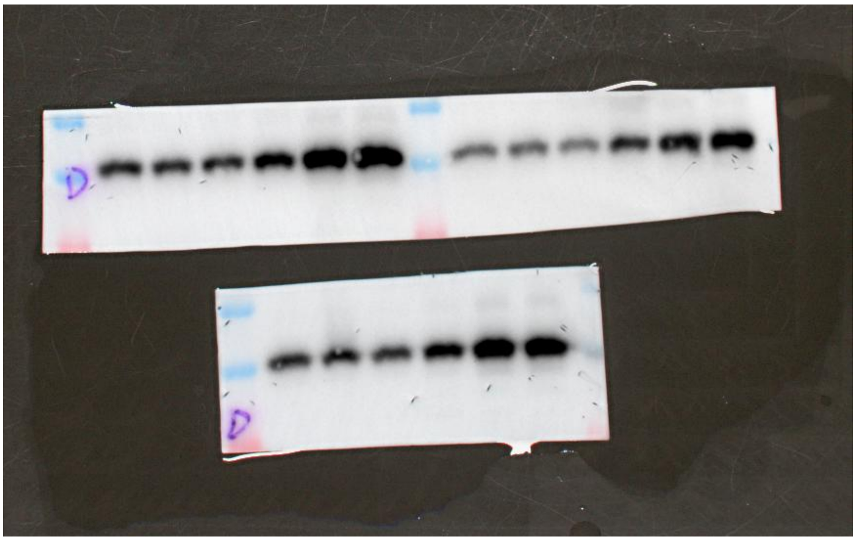

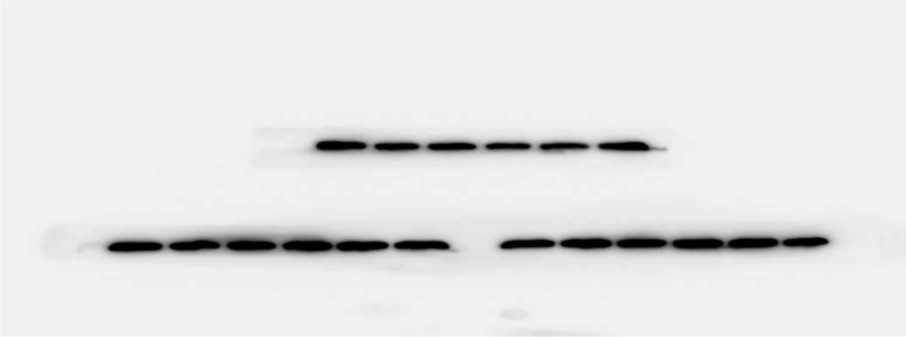

Figure 6. G

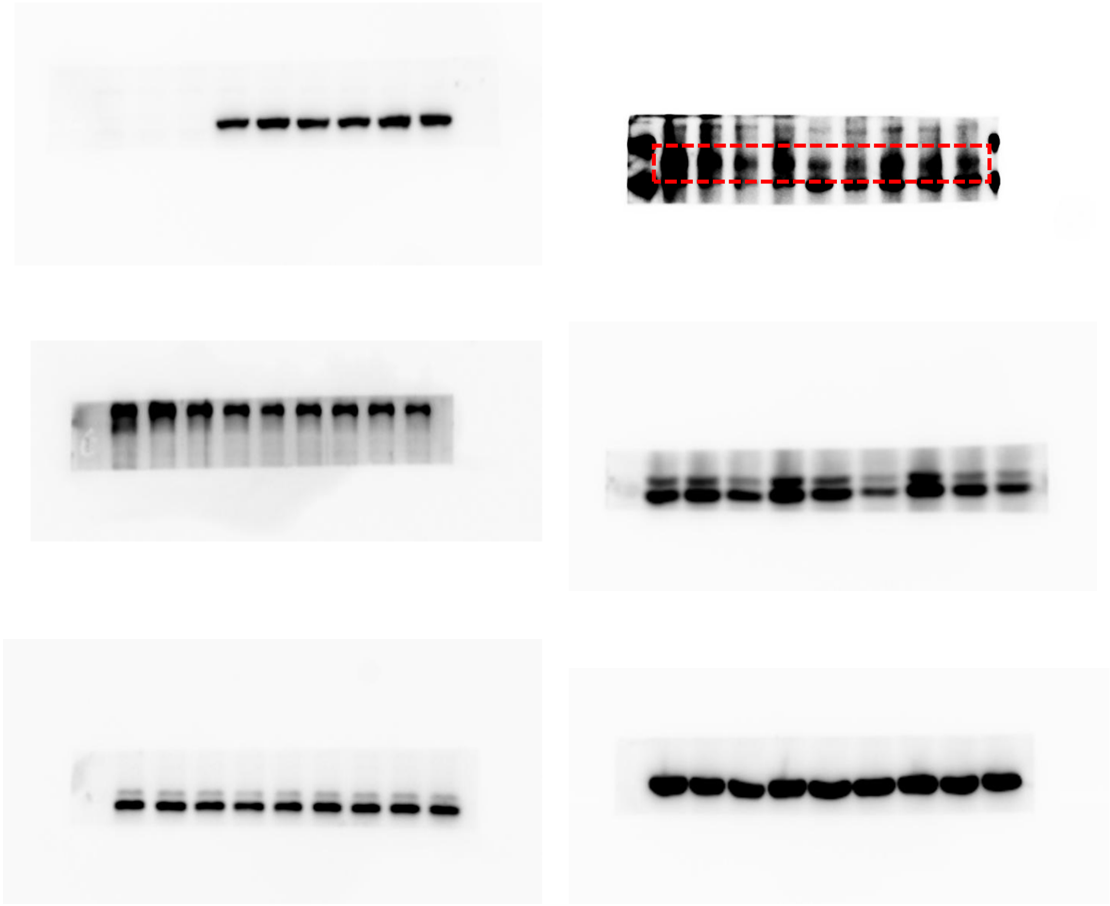

Figure 7. D

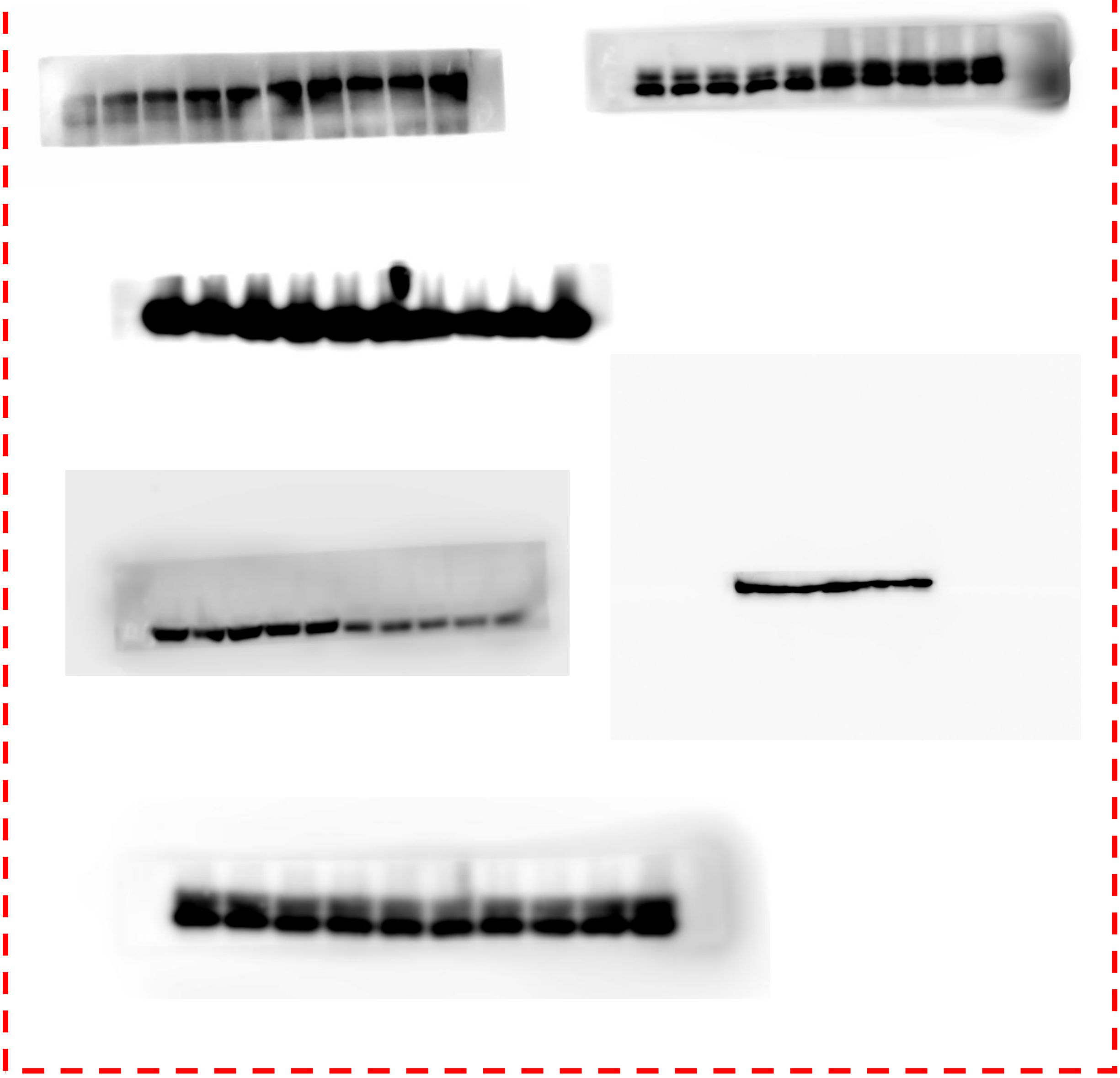

Figure 7. I

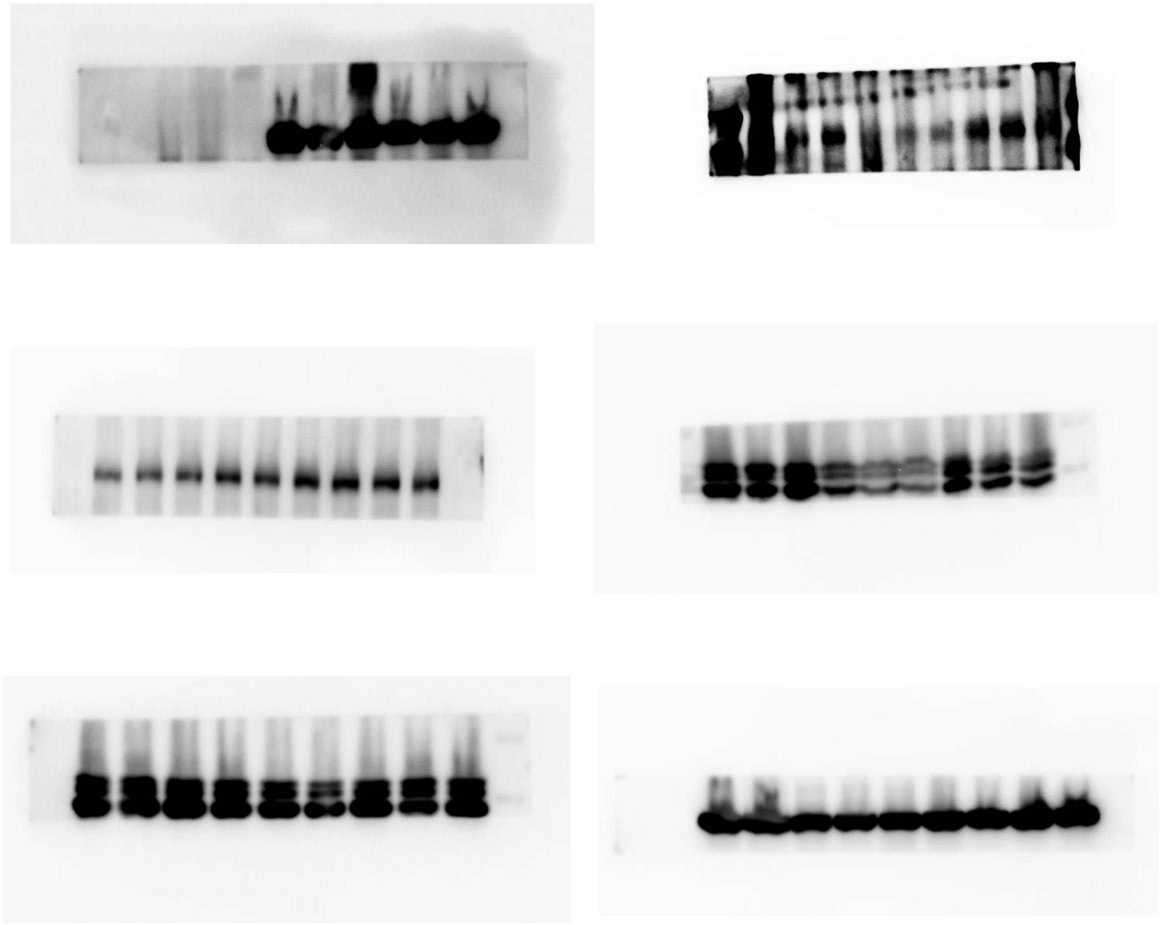

Supplement: Supplementary file 2 — Additional file 1. [file 12964_2023_1217_MOESM1_ESM.zip › 20230630-original data file.pdf]
