# Supplementary figures and images for: SUMOylation of AnxA6 facilitates EGFR-PKCα complex formation to suppress epithelial cancer growth
Source: Cell Commun Signal. 2023 Aug 1;21:189. doi: 10.1186/s12964-023-01217-x (PMC10391975; doi:10.1186/s12964-023-01217-x)

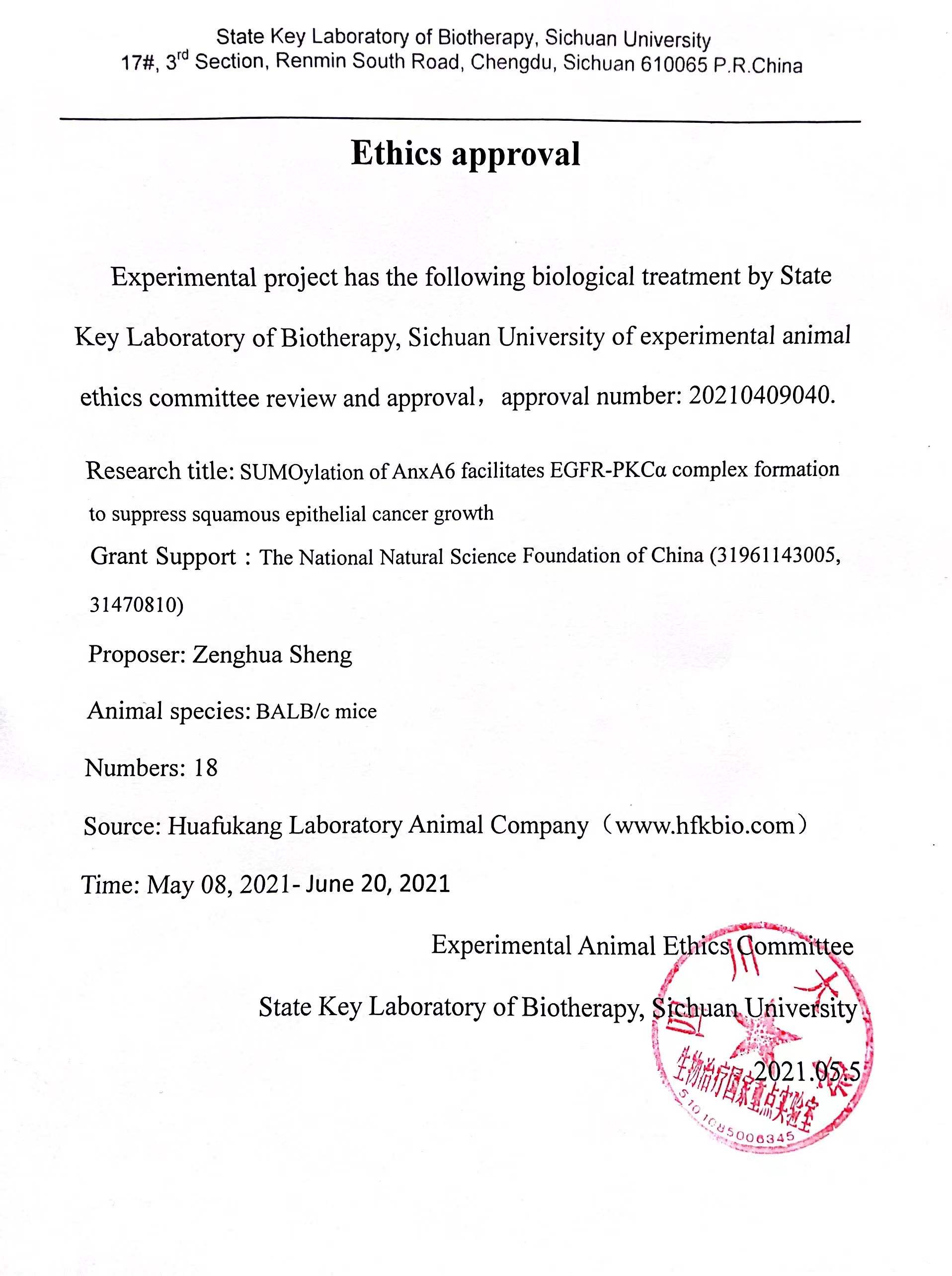

Supplement: Supplementary file 2 — Additional file 1. [file 12964_2023_1217_MOESM1_ESM.zip › Ethics approval.jpg]
