# Supplementary material for: SUMOylation of AnxA6 facilitates EGFR-PKCα complex formation to suppress epithelial cancer growth
Source: Cell Commun Signal. 2023 Aug 1;21:189. doi: 10.1186/s12964-023-01217-x (PMC10391975; doi:10.1186/s12964-023-01217-x)
Supplement: Supplementary file 2 — Additional file 1. [file 12964_2023_1217_MOESM1_ESM.zip › The track change manuscript.doc]

**SUMOylation of AnxA6 facilitates EGFR-PKCα complex formation to suppress** **epithelial cancer growth**

Zenghua Sheng^1^, Xu Cao^1^, Ya-nan Deng^1^, Xinyu Zhao^1^, Shufang Liang^1^*

^1^ Department of Biotherapy, Cancer Center and State Key Laboratory of Biotherapy, West China Hospital, Sichuan University, Chengdu, 610041, P. R. China.

*****	Correspondence: Dr. Shufang Liang, Department of Biotherapy, Cancer Center and State Key Laboratory of Biotherapy, West China Hospital, Sichuan University, No.17, Section 3 of Renmin South Road, Chengdu, 610041, China. E-mail: zizi2006@scu.edu.cn.

**Abstract**

**Background:** The Annexin A6 (AnxA6) protein is known to inhibit the epidermal growth factor receptor (EGFR)-extracellular signal regulated kinase (ERK)1/2 signaling upon EGF stimulation. While the biochemical mechanism of AnxA6 inactivating phosphorylation of EGFR and ERK1/2 is not completely explored in cancer cells.

**Methods:** Cells were transiently co-transfected with pFlag-AnxA6, pHA-UBC9 and pHis-SUMO1 plasmids to enrich the SUMOylated AnxA6 by immunoprecipitation, and the modification level of AnxA6 by SUMO1 was detected by Western blot against SUMO1 antibody. The SUMOylation level of AnxA6 was compared in response to chemical SUMOylation inhibitor treatment. AnxA6 SUMOylation sites were further identified by LC-MS/MS and amino acid site mutation validation. AnxA6 gene was silenced through AnxA6 targeting shRNA-containing pLKO.1 lentiviral transfection in HeLa cells, while AnxA6 gene was over-expressed within the Lenti-Vector carrying AnxA6 or mutant AnxA6^K299R^ plasmid in A431 cells using lentiviral infections. Moreover, the mutant plasmid pGFP-EGFR^T790M/L858R^ was constructed to test AnxA6 regulation on EGFR mutation-induced signal transduction. Moreover, cell proliferation, migration, and gefitinib chemotherapy sensitivity were evaluated in HeLa and A431 cells under AnxA6 konckdown or AnxA6 overexpression by CCK8, colony form and wound healing assays. And tumorigenicity *in vivo* was measured in epithelial cancer cells-xenografted nude mouse model.

**Results:** AnxA6 was obviously modified by SUMO1 conjugation within Lys (K) residues, and the K299 was one key SUMOylation site of AnxA6 in epithelial cancer cells. Compared to the wild type AnxA6, AnxA6 knockdown and its SUMO site mutant AnxA6^K299R^ showed less suppression of dephosphorylation of EGFR-ERK1/2 under EGF stimulation. The SUMOylated AnxA6 was prone to bind EGFR in response to EGF inducement, which facilitated EGFR-PKCα complex formation to decrease the EGF-induced phosphorylation of EGFR-ERK1/2 and cyclin D1 expression. Similarly, AnxA6 SUMOylation inhibited dephosphorylation of the mutant EGFR, thereby impeding EGFR mutation-involved signal transduction. Moreover, AnxA6 knockdown or the K299 mutant AnxA6^K299R^ conferred AnxA6 inability to suppress tumor progression, resulting in drug resistance to gefitinib in epithelial cancer cells. And in epithelial cancer cells-xenografted nude mouse model, both the weight and size of tumors derived from AnxA6 knockdown or AnxA6^K299R^ mutation-expressing cells were much greater than that of AnxA6-expressing cells.

**Conclusions:** Besides EGFR gene mutation, protein SUMOylation modification of EGFR-binding protein AnxA6 also functions pivotal roles in mediating epithelial cancer cell growth and gefitinib drug effect.

**Keywords:** Annexin A6, SUMOylation, EGFR, Gefitinib

**Background**

SUMOylation is one type of post-translational modifications (PTMs) (1) to regulate multiple cellular processes and functions. SUMOylation confers a small ubiquitin-like modifier (SUMO) protein conjugation to a lysine (K) residue of the target protein (2). The reversible attachment of a SUMO to a protein is controlled by an enzymatic reaction pathway that is analogous to the ubiquitin modification (3). SUMOylation is a dynamic process which mediates biological activities of the substrate protein including subcellular distribution, protein stability and activity, and interaction with other proteins (4). The imbalance of SUMOylation is tightly associated with cancer occurrence and progression (3-10).

The epithelial growth factor receptor (EGFR) is a cell surface glycoprotein designated the EGF receptor belonging to ErbB family of tyrosine kinase and has been shown to have an active role in a variety of tumor development (11-12). The carboxy terminal tyrosine residues of EGFR, tyrosine 1068 and tyrosine 1173, are the major sites of autophosphorylation, which occurs as a result of EGF binding. Once being activated, EGFR initiates a cascade of downstream signaling pathways including RAS/RAF/MEK/ERK and PI3K/AKT/mTOR pathways, thereby regulating cell proliferation, survival, and differentiation (13).

This high incidence of EGFR-related cancers and its association with poor clinical outcomes pave way for development of therapeutic interventions, including small-molecule tyrosine kinase inhibitors (TKIs), including the first-generation gefitinib, erlotinib and icotinib, the second-generation afatinib and dacomitinib and the third-generation osimertinib (14-15). The first generation TKIs are typically used to treat patients with high EGFR expression who carry L858R or exon 19 deletion mutations but were resisted by a second mutation T790M. Second generation or third-generation TKIs are effective against T790M EGFR. However, primary or acquired resistance to TKIs seems inevitable and severely hinders patients from getting further benefits from treatment (16-18). Interestingly, the presence or absence of some scaffold proteins like Akt kinase-interacting protein 1 and caveolin, are known to recruit positive or negative regulators of the EGFR to modulate their signal output and alter sensitivity toward drugs targeting the EGFR(19-20). Hence, as the relative amounts of scaffolds can substantially influence the strength of EGFR signaling networks, this has potential to alter TKI efficacy.

Annexin A6 (AnxA6) acts as a multifunctional scaffold protein by recruiting several signaling proteins, modulating actin dynamics and mediating the endosome aggregation or vesicle fusion in secreting epithelia during exocytosis (21). More interestingly, AnxA6 exhibits dual functions either as a tumor suppressor or oncogene in carcinogenesis (22-31), which is dependent on its recruitment of different target proteins to involve in cancer cell activities. AnxA6 displays cancer suppression effects on A431 cells by interacting with protein kinase Cα (PKCα) to reduce EGF-induced tyrosine phosphorylation of EGFR (pY-EGFR) and cycling D1 expression (23-26). Similarly, knockdown of AnxA6 enhances phosphorylation of ERK1/2 following EGF stimulation and regulates EGFR/Ras signaling pathway in HeLa cells (26). However, AnxA6 acts as a promoting factor in invasiveness of breast cancer (29-30) and acute lymphoblastic leukemia (31).

Considering a multi-faced scaffolding role of AnxA6, we speculate it exerts biological function not only through interacting with specific proteins but also by ways of PTMs. As an example of our recent findings, it has been confirmed another adaptor protein IQGAP1 is much SUMOylated in colorectal cancer. The SUMOylated IQGAP1 enhances colorectal cancer cell growth, cell migration and tumorigenesis in vitro and in vivo through activating the phosphorylation of ERK, MEK and AKT (7). However, which kind of PTMs on AnxA6 and how to regulate biological process are rarely known in cancer cells.

In this study, we have revealed AnxA6 is modified by SUMO1 in epithelial cancer cells including A431 and HeLa. SUMOylation of AnxA6 at K299 residue facilitates the binding of PKCα to EGFR and subsequently impedes the EGFR activity. Compared to the wild-type AnxA6, AnxA6 knockdown or the K299 mutant AnxA6^K299R^ up-regulates the phosphorylation level of EGFR-ERK1/2 under EGF stimulation, thereby promoting epithelial cell proliferation and migration. Meanwhile, AnxA6 SUMOylation suppresses dephosphorylation of EGFR mutations (particularly T790M and L858R double mutation), thereby impeding EGFR mutation-involved signal transduction. Moreover, the EGFR tyrosine kinase inhibitor gefitinib more effectively inhibits cell viability, clonogenic growth, and wound healing of the wild type AnxA6 cells compared to AnxA6 knockdown cells or the K299 mutant AnxA6^K299R^ cells. Collectively, AnxA6 SUMOylation plays a critical role in mediating EGFR-PKCα complex formation to suppress phosphorylation of EGFR-ERK1/2 signaling pathway, which much effectively enables gefitinib to inhibit proliferation and migration of epithelial cancer cells.

**Materials and Methods**

**Reagents and antibodies**

Cell culture media DMEM and RPMI-1640, fetal bovine serum (FBS) were ordered from Gibco. Human recombinant EGF (Catalog number:10605-HNAE) was bought from Sino Biological company. ML792 (HY-108702), SUMOylation inhibitor 2-D08 (HY-114166), gefitinib (HY-50895), puromycin (HY-B1743A) and N-ethylmaleimide (NEM) (HY-D0843), were ordered from MedChemexpress company. Slurry anti-Flag M2 affinity gel (A2220) was ordered from Sigma. The protein-A beads (161-4013) were bought from Bio-Rad. The primary antibodies included individual monoclonal antibody of Myc (ab18185, Abcam), EGFR (sc-373746, Santa Cruz), AnxA6 (sc-166807, Santa Cruz). And rabbit monoclonal antibodies against Flag (BX00086), HA (BX00069-C3), His (BX00085-C3), SUMO1(ET1606-53), UBC9(ET1610-21), pY-EGFR(1086) (ET1612-30) and p-ERK1/2 (ET1610-13), ERK1/2 (ET1601-29 ), Cyclin D1(SA38-08) were all ordered from HuaBio Company in China. The antibody of β-tubulin (TA-10, Zsbio) and β-actin (TA-09, Zsbio) was used to quantify expression of housekeeping gene β-tubulin or β-actin for comparison normalization. The IgG antibody (A7016, Beyotime) was taken as nonspecific binding control for IP performance.

**Cell culture**

HEK293T, HeLa and A431 cell lines were cultured with DMEM or RPMI-1640 medium including 10% FBS and 100 unit’s penicillin-streptomycin in a 37°C incubator with 5% CO_2_ and 95% air.

**Plasmids transfection and generation of stable cell lines**

The AnxA6 cDNA (gi71773329) was cloned into a eukaryotic expression vector pTango-zeo which contains a Flag tag, and the recombinant plasmid named pFlag-AnxA6 was verified by DNA sequencing. The mutant plasmids, including pFlag-AnxA6^K75R^, pFlag-AnxA6^K306R^, pFlag-AnxA6^K418R^, pFlag-AnxA6^K579R^, pFlag-AnxA6^K156R^,pFlag-AnxA6^K299R^ and pFlag-AnxA6^K314R^ were derived from pFlag-AnxA6 through site mutagenesis performed by Genecopoeia Company, and all mutations were confirmed by DNA sequencing. The plasmids pHis-SUMO1, pHis-SUMO2, pHis-SUMO3, pMyc-SUMO1, and pHA-UBC9 were used and stored in our laboratory (7). And the plasmids pMD2.G and pSPAX2 were provided by Beijing Tsingke Biotech Co., Ltd.

The mutant plasmid pGFP-EGFR^T790M/L858R^, with double mutations of T790 mutation to M and L858 mutation to R, was derived from pGFP-EGFR (32) through site mutagenesis performed by Beijing Tsingke Biotech Co., Ltd., which was confirmed by DNA sequencing. To construct an expression plasmid with specific shRNAs targeting AnxA6 (shRNA-AnxA6), primer pairs containing shRNA-AnxA6 sequences were mixed, annealed and inserted into the pLKO.1 Lenti vector, and the recombinant plasmid was named pLKO.1-shRNA-AnxA6.

Plasmids were transiently transfected into HEK293T, HeLa and A431 cells with the transfection reagent of Lipofectamine2000 (11668–019, Life Technologies) to observe biological effects. For generation of AnxA6-overexpressing cells, HEK293T cells are co-transfected with pMD2.G, pSPAX2, and pFlag-AnxA6 or pFlag-AnxA6^K299R^ plasmids, after transfection of 72h, 10 mL lentivirus-containing medium was collected to infect target cells, following stable cells were screened with 2 μg/ml puromycin for two weeks. Similarly, plasmids pMD2.G, pSPAX2, and pLKO.1-shRNA-AnxA6 were co-transfected into HEK293T cells to generate and screen stable AnxA6-knockdown cells with 2 μg/ml puromycin for two weeks.

**Immunoprecipitation**

2×10^7^ cells were harvested to extract total proteins to enrich the target protein by immunoprecipitation (IP) mainly according to our previous approaches (7). The supernatant of lysates with approximately containing 1-2 mg protein were incubated with 50 μl of slurry anti-Flag M2 affinity gel overnight at 4°C. To capture AnxA6 protein, 2 mg cellular supernatant was incubated with the anti-AnxA6 antibody and the protein-A beads overnight. As a negative control, the normal rabbit IgG was performed IP to eliminate the nonspecific protein binding. After washing 4 times with TBS buffer, the protein complexes were eluted with the sample-loading buffer to run SDS-PAGE for Western blot detection.

**Western blot**

Cell lysates or protein samples from IP were separated on a 7.5-12.5% SDS-PAGE gel to test the protein expression abundance by Western blot against specific antibodies. The specific primary antibodies included anti-Flag (BX00086, HuaBio), anti-SUMO1 (ET1606-53, HuaBio), anti-AnxA6 (sc-166807, Santa Cruz) and anti-β-actin (TA-09, Zsbio) antibodies. The PVDF membranes were blocked in 5% nonfat milk at room temperature for 1.5 h and then incubated with specific primary antibodies at 4℃ overnight. The corresponding secondary antibody was incubated with the PVDF membrane for 1h at room temperature. The protein bands were visualized by enhanced chemiluminescence (Millipore, USA) and analyzed using ImageJ software.

**Cell proliferation**

Cell proliferation was analyzed by CCK8 according to our previous methods (7,33). After cell transfection for 24 h, 2 ×10^3^ Hela or A431 cells/well were seeded into a 96-well plate to incubate for up to 120h with medium replenished every 48 h. Cells were cultured in DMEM or RPMI-1640 containing 0.1% (v/v) FBS and 100 ng/ml EGF to measure cell proliferation using CCK8 approach. Moreover, colony-forming assays were performed as described (23), and 1000 cells were grown in 6-well plates with 0.1% (v/v) FBS and 100 ng/ml EGF treated for 14 days.

**Wound healing assays**

The A431 cells were seeded into a 6-wells plate at an appropriate concentration. After being transfected with pFlag-AnxA6 or pFlag-AnxA6^K299R^ plasmids for 24 h, then a scratch was made across the center of each well (7, 14)^.^ And Cells were washed with PBS three times to remove the detached cells. After cells were allowed to grow for 36 h in RPMI-1640 containing 0.1% (v/v) FBS and 100 ng/ml EGF, wound margins were photographed and cell migration was observed under an inverted microscope.

**Liquid chromatography with tandem mass spectrometry (LC-MS/MS)**

HEK293T cells overexpressing AnxA6 and SUMO1^T95K^, were collected to enrich SUMO1-tagging AnxA6 by IP (34). The sample was eluded for digesting by Lys-C enzyme, and peptides were identified by liquid chromatography-tandem mass spectrometry(LC-MS/MS) on an easy nano-LC1000 HPLC system (Thermo Scientific, San Jose, CA) and a Q-Exactive mass spectrometry (Thermo Scientific, San Jose, CA)(35-36). Survey scan ranged 300-1800 m/z at a resolution of 70,000. After full scan, top 10 MS fragments were selected for higher-energy collisional dissociation. Isolation window was acquired at a resolution of 17,500 with an isolation window of 1.6 m/z. The MS/MS scan was 1 ×10^6^ with a maximum injection time of 20 ms, and that for the MS/MS scan was 1×10^5^ with a maximum injection time of 100 ms.The data were searched by MaxQuant search engine (version 2.2, Matrix Sciences, London, UK). Parameters were chosen as follows: the human Swiss-Prot database (version 2019.12), up to two missed cleavage sites for Lys-C, peptide mass tolerance of 7 ppm, and fragment mass tolerance of 0.5 Da for the higher-energy collisional dissociation (HCD)(36). Carbamidomethylation of cysteine was specified as a fixed modification, whereas oxidation (M), acetyl (protein N-term), and KGG (only for indicating SUMOylation) were defined as variable modifications. The false discovery rates (FDRs) of peptide and protein were set to 0.01 FDR. + 2 as default charge state of each peptide. And at least one unique peptide of a protein successfully detected was considered to be acceptable.

**Xenograft tumor model**

All mouse experiments on BALB/c mice with 5-week-old were approved and conducted by the Institutional Animal Care and Treatment Committee of Sichuan University, China. A suspension containing 5 × 10^6^ HeLa or AnxA6-knockdown containing HeLa cells was subcutaneously injected into the right flanks of the 5-week-old male BALB/c nude mice (n = 5) to construct tumor models respectively. Similarly, 5 × 10^6^ cells stably expressing Flag-AnxA6 or Flag-AnxA6^K299R^ suspended in 100μl PBS were harvested and injected into the right flanks of 5-week-old male BALB/c nude mice (n = 5) individually. About 6 days after HeLa cell injection or 9 days after A431 cell injection, tumor volumes were measured every 3 days using a vernier caliper and calculated as follows: V = (length × width × height × 0.5) mm^3^. At 30 days of inoculation, mice were sacrificed, and tumors were isolated, photographed, weighed and collected for further examination.

**Immunohistochemistry**

Immunohistochemistry (IHC) staining and quantitative scoring were performed as previously described (37-38). The primary anti-Ki67 antibody was diluted at 1:800 to perform IHC, and all samples were visualized under a Leica DM 2000 microscope.

**Statistics analysis**

The significance of differences was determined using the Students test. All quantitative data were expressed as means ± S.D. p<0.05 was regarded as a significant difference.

**Results**

1. **AnxA6 is modified by SUMO1 conjugation**

To determine which type of SUMO molecules conjugates with AnxA6, three kinds of exogenous His-tagging SUMO plasmids (pHis-SUMO1, pHis-SUMO2 and pHis-SUMO3) were simultaneously co-transfected with pFlag-AnxA6 and pHA-UBC9 plasmids into HEK293T cells to compare the SUMOylated AnxA6 levels. The Flag-tagging AnxA6 protein was captured from total cellular proteins by immunopreciptation (IP) using anti-Flag antibody beads. The most obvious SUMO-tagging AnxA6 bands with a molecular weight of 95kDa (the expected normal size of AnxA6 is 75 kDa) appeared in cell co-transfection of pHis-SUMO1 and pHA-UBC9 plasmids (Fig. 1A, Lane 2). Similarly, we also transiently transfected pFlag-AnxA6 along with three kinds of exogenous His-tagging SUMO plasmids in HEK293T cells, and following the purification of His-tagging SUMO conjugates with Ni^2+^-NTA agarose beads. The most obvious SUMO-tagging AnxA6 band was also confirmed with anti-Flag antibody in the cells co-transfected with SUMO1, but not in the cells transfected with SUMO2 or SUMO3 (Fig. 1B, Lane 2). Taken together, our data indicated AnxA6 was obviously modified by SUMO1 conjugation.

Next, we further detected AnxA6 SUMOylation change level in response with cell treatment by chemical SUMOylation inhibitors. The exogenous expressing AnxA6 protein was obviously SUMOylated by being transiently transfected pFlag-AnxA6 plasmids in HEK293T cells (Fig. 1C, Lane 2). As expected, the abundance of SUMOylated AnxA6 was significantly enhanced upon UBC9 stimulation (Fig. 1C, Lane 3). While under the treatment of 150 μM inhibitor 2-D08 for 24h, SUMOylated AnxA6 was significantly decreased in spite of UBC9 existence (Fig. 1C, Lane 4), which was due to 2-D08 interference for the binding of UBC9 with SUMO1(10). In addition, we also observed AnxA6 SUMOylation was lost due to another SUMOylation inhibitor ML-792 treatment (Fig. 1D, Lane 4). Collectively, these data demonstrated that AnxA6 was modified with SUMO conjugation.

2. **K299 is** **a** **predominant SUMOylation site of AnxA6**

Next, the pFlag-AnxA6 and pHis-SUMO1^T95K^ (T95 mutation to K) plasmids were simultaneously co-transfected into HEK293T cells to identify the AnxA6 SUMOyaltion site by MS/MS analysis. The obvious His-SUMO1^T95K^-tagging AnxA6 band was verified by Western blot against anti-His antibody (Fig. 2A). Using the mutant SUMO tagging method, K156, K299 and K314, which were non-consensus SUMOylation sites, were identified as potential predominant SUMOylation sites in AnxA6 by MS/MS analysis. The MS/MS spectra of the target peptides, including DAYERDLEADIIGDTSGHFQK^156^, YEK^299^SLYSMIK and NDTSGEYK^314^K of AnxA6, were obtained using HCD scan, which contained the SUMO-modified K156, K299 and K314 residue respectively (Fig. 2B). The matching parameters of MS/MS identification were summarized in Figure 2C.

As known, SUMOylation site within a protein typically occurs at the K residue that is located within the consensus sequence ψKxD/E, where “ψ” is a hydrophobic residue and “x” means any sort of amino acid residue (3). Totally, four potential consensus SUMOylation sites including K75, K306, K418, and K579 of AnxA6 were predicted with high-ranking scores based on bioinformatics analysis using one online software GSP-SUMO (http://sumosp.biocuckoo.org/) (Fig. 2D).

To confirm the SUMOylation site on AnxA6, we constructed seven K-residue mutated plasmids, each with a single K point mutation, to validate SUMOylation level change of AnxA6.Compared with the other K-residue mutations including K75, K156, K306, K314, K418 and K579, the single site mutation of K299R greatly impaired AnxA6 SUMOylation level (Fig. 2E-Lane 7, 2F-Lane 8).

We further monitored SUMOylation level of the wild-type AnxA6 and the mutant AnxA6^K299R^ under SUMO1 overexpression condition. Compared with the wild-type AnxA6 (Fig. 2G, Lane 2), the SUMOylation of AnxA6^K299R^ mutant was most obviously reduced under co-transfecting with pSUMO1 plasmid in HEK293T cells (Fig. 2G, Lane 4). Taken together, these findings indicated that K299 was a key SUMOylation site of AnxA6.

**3. AnxA6 SUMOylation** **inhibits** **EGFR/ERK pathway by stimulating PKCα binding to EGFR**

AnxA6 acts as a tumor suppressor through inhibiting EGFR/ERK pathway (23-24,26), therefore we would explore whether SUMOylation of AnxA6 is involved in EGFR/ERK signaling regulation. As mentioned as Fig. 1B-D, the degree of AnxA6 SUMOylation was enhanced with the increase of UBC9 expression, while AnxA6 SUMOylation was decreased even lost under SUMOylation inhibitor ML-792 treatment. Thus, on the condition of loading equal amount of Flag-AnxA6, we compared changes of cellular EGFR/ERK signaling upon EGF stimulation under increasing UBC9 expression or ML792 inhibitor treatment.

Cells were serum-starved for overnight and then stimulated with EGF for 5 min to detect EGFR/ERK signaling response. The pY-EGFR and p-ERK1/2 levels were further decreased even lost along with the co-transfection of pHA-UBC9 and Flag-AnxA6 plasmids (Fig. 3A, Lane 5), whereas AnxA6 deSUMOylation by the inhibitor ML792 treatment abolished its inhibition of EGFR/ERK phosphrylation under EGF stimulation (Fig. 3B, Lane 5). Meanwhile, the exogenous expression of wild-type AnxA6 (Fig. 3C-D, Lane 4), but not mutant AnxA6^K299R^ (Fig. 3C-D, Lane 6), really inhibited pY-EGFR and p-ERK1/2 signaling compared to the endogenorus HeLa and A431 cells (Fig. 3C-D, Lane 2).

Our above results have proven the new concept that AnxA6 SUMOylation enhances its suppressive effect on EGFR/ERK phosphorylation in HeLa and A431 cells, so next we want to get insight into underlying the molecular mechanisms. Considering AnxA6 is a scaffold for promoting EGFR-PKCα interaction that is closely related with inactivation of EGFR/ERK pathway, we speculate that AnxA6 SUMOylation affects EGFR-PKCα complex formation. To validate the possibility, cell lysates were subjected to perform IP with EGFR antibody to enrich the EGFR-binding complex. The EGFR-binding PKCα was increased under EGF stimulation in AnxA6-overexpressing A431 cells (Fig. 3E, Lane 4) compared with that of AnxA6^K299R^-overexpressing A431 cells (Fig. 3E, Lane 6), which indicated that AnxA6 SUMOylation facilitated EGFR-PKCα complex formation. Interestingly, our data also showed that EGFR was susceptible to bind wild-type AnxA6 than mutant AnxA6^K299R^ protein (Fig. 3E, Lanes 3-4 versus Lane 5-6), which supports AnxA6 SUMOylation enhances its binding with EGFR and subsequently promotes the association of PKCα with EGFR.

Indeed, we observed that an increased binding of AnxA6 with EGFR in HeLa cells correlated with an upregulation of AnxA6 SUMOylation level, whereas the binding of PKCα to AnxA6 was not changed under UBC9 overexpression or ML792 treatment (Fig. 3F, Lane 3-4). To further validate this finding, we examined the effect of the K299 site mutant Flag-AnxA6^K299R^ on the binding of EGFR and PKCα. Indeed, AnxA6 overexpression significantly enhanced the amount of EGFR that was enriched by IP against Flag-AxA6 antibody, especially in the condition of EGF stimulation (Fig. 3G, Lane 3). In contrast, AnxA6^K299R^ overexpression had no obvious effect on its interaction with EGFR in A431 cells (Fig. 3G, Lane 6). Moreover, PKCα enriched by IP with Flag-AnxA6 antibody was a similar amount as to that bound with the Flag-AnxA6^K299R^ (Fig. 3G, Lanes 2-3 versus 4-5)，which supported the conclusion that SUMOylation of AnxA6 did not mediate AnxA6-PKCα interaction. Collectively, these results indicate that AnxA6 SUMOylation promotes its binding with EGFR to increase EGFR-PKCα complex formation, thereby leading to inactivation of the EGFR/ERK phosphorylation signaling pathway.

**4.** **AnxA6 SUMOylation** **suppresses activation of EGFR** **mutation-induced** **signal transduction**

EGFR mutations (particularly T790M and L858R double mutation) are known as a major cause of EGFR signaling hyperactivation and EGFR-targeted drug acquired resistance in many cancers, especially in non-small cell lung carcinoma（NSCLC）(39-40). We first investigated influence of EGFR mutation on AnxA6-mediated phosphorylation of EGFR and ERK1/2. We silenced the expression of AnxA6 in HeLa cells through the pLKO.1 lentiviral system with the specific AnxA6-targeting shRNA, and then transiently transfected pGFP-EGFR^T790M/L858R^ plasmids into cells to generate a site double-mutated EGFR^T790M/L858R^. When AnxA6 expression was significant knockdown, the pY-GFP-EGFR^T790M/L858R^, pY-EGFR and p-ERK1/2 levels were further enhanced upon EGF stimulation (Fig. 4A), which indicated that AnxA6 also involved in EGFR mutation-induced signal transduction.

We further revealed whether AnxA6 SUMOylation had influence on mutant EGFR signal transduction. As shown in Figure 4B, the wild-type AnxA6 overexpression significantly suppressed the pY-GFP-EGFR^T790M/L858R^, pY-EGFR and p-ERK1/2 levels. In contrast, the mutant AnxA6^K299R^ overexpression was unable to inhibit the phosphorylation levels of these proteins in A431 cells with transiently transfection of pGFP-EGFR^T790M/L858R^ plasmids (Fig. 4B, Lanes 2 versus 4). Moreover, the GFP-EGFR^T790M/L858R^, as same as EGFR, increased its binding to PKCα and AnxA6 in AnxA6-expressing A431 cells upon EGF stimulation, but not in AnxA6^K299R^-expressing A431 cells (Fig. 4C, Lane 2 versus 4).

Meanwhile, we enriched Flag-AnxA6^K299R^-binding protein complex by IP against Flag antibody and analyzed AnxA6^K299R^-binding EGFR level by Western blot. As expected, the wild-type AnxA6 exhibited an evidently increased binding capability with GFP-EGFR^T790M/L858R^ than the mutant AnxA6^K299R^ under EGF-treated conditions (Fig. 4D, Lane 2 versus 3). Together, these data revealed that AnxA6 SUMOylation inhibited phosphorylation of EGFR^T790M/L858R^, thereby impeding EGFR mutation-involved signal transduction.

**5. AnxA6 SUMOylation** **impedes** **cell proliferation and migration by inactivating pY-EGFR and p-ERK1/2**

Next, we clarified biological function of AnxA6 in cancer cell proliferation and migration. We first established AnxA6-knockdown cell model in HeLa by stably expressing shRNA targeting AnxA6, named HeLa-AnxA6(KD). Consistent with published data (23), the pY-EGFR and p-ERK1/2 were significantly enhanced in HeLa-AnxA6(KD) cells after starvation following EGF stimulation (Fig. 5A, Lane 2 versus 4). And the AnxA6 knockdown also inhibited formation of EGFR-PKCα complex upon EGF stimulation (Fig. 5A, Lane 2 versus 4).

Moreover, the CCK8, colony-forming and wound healing assays were applied to detect AnxA6 knockdown-mediated cell proliferation and migration. In comparison with HeLa, cell growth and migration upon EGF induction were significantly promoted in HeLa-AnxA6(KD) cells (Fig. 5B-D).Taken together, these results indicated that AnxA6 impedes cell proliferation and migration in response to EGF stimulation by inactivating pY-EGFR and p-ERK1/2.

We further tested AnxA6 SUMOylation influences on cell proliferation and migration. A431 stable cell lines were generated by using lentiviral infections with the Lenti-Vector carrying wild-type AnxA6 or mutant AnxA6^K299R^ plasmid, and the corresponding two stable cell clones were named as A431-AnxA6 and A431-AnxA6^K299R^. Consistent with the above conclusions, the pY-EGFR and p-ERK1/2 in A431-AnxA6^K299R^ cells were significantly increased than that of A431-AnxA6 cells upon EGF stimulation (Fig. 5E, Lanes 2 versus 3). Moreover, the level of AnxA6 SUMOylation and AnxA6-binding EGFR in A431-AnxA6^K299R^ cells was much less than that in A431-AnxA6 cells (Fig. 5E, Lanes 2 versus 3).

In comparison with A431 cells, cell growth and migration upon EGF induction were lagged under the ectopic expression of wild-type AnxA6 and mutant AnxA6^K299R^ in A431 cells (Fig. 5F-H). It was noted cell proliferation and migration of A431-AnxA6^K299R^ were relatively improved than A431-AnxA6 cells, which indicates SUMOylation of AnxA6 at K299 site is helpful to attenuate cell growth and migration.

To further confirm AnxA6 SUMOylation-mediated inhibition of the phosphorylation of EGFR/ERK signaling, we measured cell cycle regulator cyclin D1, a downstream target of EGFR/ERK signaling pathway (Fig. 5I). In A431 cells, upon with EGF stimulation, the cyclin D1 level was significantly increased from 0.5h to 6h (Fig. 5I, the top). By comparison, in the EGF-stimulated A431-AnxA6 cells (Fig. 5I, the Middle), the increase degree of cyclin D1 was obviously slower within 2h to 6h than that of A431-AnxA6^K299R^ cells (Fig. 5I, the bottom). Thus, the K299R mutation of AnxA6 impaired its [activity](../../../../C:/Users/95172/AppData/Local/youdao/dict/Application/8.5.1.0/resultui/html/index.html#/javascript:;) in suppressing EGF-induced cyclin D1 expression, which further illustrated that AnxA6 SUMOylation inhibited phosphorylation of EGFR/ERK signaling transduction.

**6.** **AnxA6 SUMOylation** improve**s gefitinib efficacy of suppressing A431 cell proliferation and** **migration**

We next evaluated whether AnxA6 co-operated with gefitinib to inhibit cancer cell proliferation and migration. HeLa and HeLa-AnxA6(KD) cells were incubated with 0-40µM gefitinib for 48h, then cell viability was determined by CCK8 assay. The difference in cell viability between HeLa and HeLa-AnxA6(KD) at 20μM gefitinib incubation was the largest of all the concentrations (Fig. 6A). Under 20μM gefitinib treatment, 77.1±5% HeLa-AnxA6(KD) cells kept cell viability, while 52.5±5% HeLa cells were alive (Fig. 6A). In addition, 20 µM gefitinib more effectively inhibited cell migration of HeLa cells compared to HeLa-AnxA6(KD) cells (Fig. 6C). These results indicated AnxA6 co-operated with gefitinib-mediated inhibition of cell growth and migration.

Considering AnxA6 improving anti-growth and anti-migratory effect of gefitinib, we speculated that AnxA6 SUMOylation enhanced gefitinib efficacy to reduce epithelial cancer cell proliferation and migration. Similarly, A431, A431-AnxA6 and A431-AnxA6^K299R^ cells were incubated with 0-30µM gefitinib for 24h to allow cell proliferation, and cell viability was determined by CCK8 assays. The difference in cell viability between A431-AnxA6 and A431-AnxA6^K299R^ at 10μM gefitinib was the largest of all the concentrations (Fig. 6D). Under 10μM gefitinib treatment, 65.1±5% A431-AnxA6^K299R^ cells kept cell viability, while 55.1±5% A431-AnxA6 cells were alive (Fig. 6D). In addition, 2.5 or 5μM gefitinib led to less colony formation in A431-AnxA6 cells compared to A431-AnxA6^K299R^ cells or A431 cells (Fig. 6E).

Moreover, the wound healing assays also shown that gefitinib more effectively inhibited A431-AnxA6 cell migration compared to A431-AnxA6^K299R^ cells (Fig. 6F). These results suggested that AnxA6 SUMOylation enhanced the efficacy of gefitinib to reduce squamous epithelial cancer cell proliferation and migration.

We further investigated EGFR/ERK signaling changes between A431-AnxA6 and A431-AnxA6^K299R^ cells with 0-10µM gefitinib treatment for 24h. The pY-EGFR and p-ERK1/2 in A431-AnxA6 cells were significantly reduced than the A431-AnxA6^K299R^ cells (Fig. 6G), which further demonstrated that AnxA6 SUMOylation cooperated with gefitinib to inhibit EGFR/ERK phosphorylation signaling.

**7. AnxA6 SUMOylation inhibits tumor growth of nude mouse model.**

Finally, we evaluated AnxA6 anti-tumor effect in xenograft nude mouse model. 5×10^6^ HeLa cells or HeLa-AnxA6(KD) cells were inoculated into the right flank of nude mice. About 6 days after injection, the tumors in each group were obviously palpable with 50 mm^3^, and subsequently the tumor size was totally monitored for 6 times from then on. At 21 days of inoculation, AnxA6 knockdown in HeLa cells significantly promoted tumorigenesis (Fig. 7A) and tumor growth (Fig. 7B). A larger tumor volume with 435.1±185.6mm^3^ (Fig. 7B) and tumor weight with 0.22±0.08g (Fig. 7C) was respectively observed in HeLa-AnxA6(KD)-injected mouse than HeLa-xenografted tumor with 204.0±63.9mm^3^ volume and 0.09±0.03g weight. In addition, compared with the HeLa-AnxA6(KD) derived tumors, levels of pY-EGFR and p-ERK1/2 in HeLa- xenografted tumors were reduced (Figure 7D).

Similarly, AnxA6 SUMOyaltion-inhibited epithelial cancer development and molecular events were further confirmed in A431-xenograft mice. We subcutaneously injected A431 cells, which stably expressing either AnxA6 or AnxA6^K299R^, into BABL/c mice to observe tumor growth. About 9 days after injection, the tumors in each group were obviously palpable with 100mm^3^, and subsequently the tumor size was totally monitored for 8 times from then on.

At 30 days of inoculation, we observed that both the wild type AnxA6 and mutant AnxA6^K299R^ dramatically inhibited the subcutaneous growth of A431 cells (Fig. 7E-G). However, the A431-AnxA6^K299R^ derived tumor, with 613.8±113.8mm^3^ and 0.31±0.065g, was much greater than the A431-AnxA6 inoculated tumor with 324.9±107mm^3^ and 0.15±0.06g (Fig. 7F-G). In addition, the A431-AnxA6 derived tumors exhibited Ki67 downregulation that the A431-AnxA6^K299R^ xenografted tumors (Fig. 7H).

Moreover, we also validated EGFR/ERK signaling changes between AnxA6 and AnxA6^K299R^-expressing tumor tissues from A431-xenograft nude mice. Compared with the A431-AnxA6^K299R^ tumors, levels of pY-EGFR and p-ERK1/2 in the A431-AnxA6 tumors were reduced (Figure 7I), and the conclusion was consistent with the data in vitro. Together, there data indicated SUMOylation-deficient AnxA6 had lost tumor-suppressor activity in vivo.

**Discussion**

AnxA6 is a member of a conserved superfamily of Ca^2+^-dependent membrane-binding annexin proteins to play multiple biochemical functions. The expression levels of AnxA6 are closely associated with melanoma, cervical cancer, epithelial carcinoma, breast cancer, gastric cancer, prostate cancer, acute lymphoblastic leukemia, chronic myeloid leukemia, large-cell lymphoma, and myeloma(41-42). And AnxA6 is also a potential candidate target for antibody-mediated inhibition of cancer (43). Numerous previous studies have focused on AnxA6 biological function by manipulating protein levels using knockout or knock-in methods. However, the PTMs of AnxA6 are rarely reported. In current study, we have identified AnxA6 SUMOylation with the K299 residue by MS/MS through introducing a T95K point mutation into the C-terminus of SUMO1 to facilitate catching SUMOylayted peptides (44). The T95K mutant SUMO tagging method does not affect the function of SUMO-1, but generates a new cutting site that leaves a Gly-Gly signature on the SUMOylated peptides after Lys-C digestion (44). This is an efficient MS identification method for SUMOylation site.

SUMO is a highly conserved molecule to conjugate to the K residue of a substrate protein in the PTM process. Numerous studies indicate that SUMOylation attenuates or escalates biological activities of the substrate protein (7,45-47), which is dependent on specific conditions including disease types, the interacting partners with the substrate protein, and the protein complex-regulated signal pathway. For instance, large tumor suppressor 1,  the core to mediate Hippo growth-inhibitory signaling pathway, is found to be modified by SUMO1 at K751 residue and attenuates its kinase activity and tumor-suppressor functions (46). Conversely, the adaptor protein Grb2 is SUMOylated by SUMO1 at K56, which increases the formation of Grb2-Sos1 complex and subsequently enhances its oncogene functions (47). In this study, our results support that AnxA6 SUMOylation at K299 residue facilitates the binding of PKCα to EGFR and subsequently enhances its tumor-suppressor activity in vitro and in vivo (Fig. 8).

The endogenous AnxA6 is absent in EGFR-overexpressing A431 cells, while a high level of AnxA6 is detectable in HeLa cells with moderate expression of EGFR(23,42). And A431 cell belong to a model cells derived from squamous cell epithelia that retain the basic characteristics of the transformed phenotype (42), therefore these cells are a well-recognized model for studying action mechanism of AnxA6 regulation on EGFR activity and the testing of pharmacological drugs targeting EGFR(14,23-26). Several previous reports uncover the scaffolding/targeting function of AnxA6 for the PKCα (23,26), both negative regulators of the EGFR-ERK pathway, probably contributes to reduce proliferation/migration of epithelial cancer cells (14, 23-24,26). And the cyclin D1, a downstream target molecule of EGFR-ERK pathway, is inhibited due to AnxA6 overexpression in EGF-induced A431 cells (23-24). Consistently, our study shows that overexpression of Flag-AnxA6, but not the SUMO-deficient mutant Flag-AnxA6^K299R^ or control pFlag vector, the most markedly suppresses cell proliferation, migration and reduced cyclin D1 expression in the A431 cells with EGF stimulation. Noticeably, the pY-EGFR and p-ERK1/2 are significantly reduced in AnxA6 wild type cells compared with AnxA6^K299R^ mutant cells. But the mutant AnxA6^K299R^ still have effective inhibition of pY-EGFR and p-ERK1/2, which suggests that other AnxA6 SUMOylation sites might be involved in the regulation of this pathway. Moreover, it's worth noting that Ca^2+^ is necessary for AnxA6 activation (14, 23-24,26). The cytosolic AnxA6 constitutively binds to PKCα in physiological state, while EGF stimulation increases intracellular Ca^2+^, which promotes the AnxA6-induced membrane recruitment of PKCα to EGFR in A431 cells (24,26,41). In this study, we discover that the formation of EGFR-PKCα complex is finely regulated by SUMO1 modification at K299 of AnxA6. In particular, our results reveal that the SUMO1 modification of AnxA6 facilitates AnxA6 binding with EGFR other than PKCα, ultimately attributing to the formation of EGFR-PKCα complex in response to extra-cellular EGF stimulation.

In addition to gene amplification of EGFR, activating mutations from EGFR are also one of the most common targetable oncogenic drivers in multiple cancers. The exon 19 deletions and L858R mutations are known as "classical" mutations, account for 85-90% of the total EGFR  mutations, whereas approximately 10% of patients have uncommon EGFR mutations including S768I, T790M, C797S and L861Q codons, and exon 20 insertions (48-51). And mutations in the EGFR, especially the T790M/L858R double mutation, have made cancer treatment more difficult (51). In this study, our results show that AnxA6 knockdown or K299 mutation of AnxA6 upregulates the phosphorylation level of exogenous expressing EGFR^T790M/L858R^ in HeLa and A431 cells, which implicates AnxA6, especially AnxA6 SUMOylation exerts inhibiting phosphorylation signaling from EGFR and mutant EGFR.

So far, the EGFR inhibition has been established a major therapeutic target in cancer therapy, particularly for tumors of breast, cervix, ovaries, kidney, esophagus, prostate and non-small-cell lung carcinoma and head and neck tumors (15-18). EGFR-TKIs, such as gefitinib, erlotinib, afatinib, dacomitinib and osimertinib, have improved the outcomes of EGFR-dependent cancers for many patients (52). However, patients eventually experience the rapid acquisition of resistance due to various mechanisms, such as compensatory activation of signaling effectors downstream of EGFR and other growth factor receptors and secondary mutations in EGFR (53). Thus, identifying an efficient therapeutic target to restore gefitinib sensitivity and improve tumor control in patients is an urgent need. Several recent studies have revealed that AnxA6 regulated the subcellular location of PKCα, with consequences not only for the activity, but also the localization and trafficking of PKCα interaction partners, including EGFR, which could ultimately also affect susceptibility for drugs targeting the EGFR-TKI domain (14,54). Indeed, our findings partly support this conclusion that AnxA6 SUMOylation enhances gefitinib efficacy to reduce A431 cell growth and migration, which indicates that AnxA6 SUMOylation may be an underlying novel mechanism for the EGFR-TKI resistance in many cancers.

Generally, we have revealed a previously unreported PTM form and molecular mechanism of AnxA6 SUMOylation in enhancing EGFR-PKCα complex formation to suppress phosphorylation of EGFR-ERK1/2 signaling pathway, which more effectively enables gefitinib to inhibit proliferation and migration of EGFR-overexpressing epithelial cancer cells.

**Conclusions**

In conclusion, we have revealed the modification of AnxA6 by SUMO1 conjugation to escalate its functions especially involving in dephosphorylation of EGFR-ERK1/2 signaling, and AnxA6 SUMOylation at K299 residue facilitates the binding of PKCα to EGFR and subsequently impedes EGFR activity, thereby inhibiting cell proliferation, migration, and the xenograft tumor growth of epithelial cancer cells, as well as improving gefitinib drug sensitivity. Moreover, AnxA6 SUMOylation can also inhibit the activating EGFR mutations signal transduction. Together, our novel findings indicate besides EGFR gene mutation, protein post-translational modification of the EGFR-binding protein AnxA6 also functions pivotal roles mediating cancer cell growth and EGFR inhibitor drug effect.

**Abbreviations**

Co-immunoprecipitation (co-IP); Western blot (WB); Post-translational modifications (PTMs); the small ubiquitin-like modifier (SUMO); lysine (K); arginine (R); Annexin A6 (AnxA6); epidermal growth factor receptor (EGFR); extracellular-signal-regulated kinase (ERK)1/2; epidermal growth factor (EGF); tyrosine phosphorylation of epidermal growth factor receptor (pY-EGFR); Non-small cell lung carcinoma（NSCLC; Tyrosine kinase inhibitors (TKIs); phosphorylation of extracellular-signal-regulated kinases ERK1/2 (p-ERK1/2); immunoprecipitated (IP); fetal bovine serum (FBS); higher-energy collisional dissociation (HCD); protein kinase Cα (PKCα); p120GTPase-activating protein (p120GAP); Immunohistochemistry (IHC).

**Declarations**

**Ethics approval and consent to participate**

The animal study were performed in accordance with the guidelines of Sichuan University and approved by the Animal Care Committee of Sichuan University (Chengdu, China).

**Consent for publication**

Not applicable.

**Data availability**

The MS proteomics data and the search data by MaxQuant is available in the Mendeley Data (https://data.mendeley.com/datasets/rdw3fk6cng/1).

**Competing interests**

The authors declare that they have no conflicts of interest with the contents of this article.

**Funding**

This work was supported by the National Natural Science Foundation of China (31961143005, 31470810), and Foundation of Chengdu Science & Technology Committee (2020-GH03-00004-HZ)*.*

**Author contributions**

SZ carried out experiments and wrote the manuscript draft. CX performed experiments. DY and ZX analyzed MS data. LS supervised the study, instructed experiments and revised manuscript. All authors participated in the preparation of the manuscript, read and approved the final manuscript.

**Acknowledgements**

Not applicable.

**References**

1. Sheng Z, Zhu J, Deng YN, Gao S, Liang S. SUMOylation modification-mediated cell death. Open Biol. 2021; 11: 210050.
2. Du L, Liu W, Rosen ST. Targeting SUMOylation in cancer. Curr Opin Oncol. 2021; 33:520-525.
3. Yang Y, He Y, Wang X, Liang Z, He G, Zhang P, et al. Protein SUMOylation modification and its associations with disease. Open Biol. 2017; 7:170167.
4. Zhu X, Qiu C, Wang Y, Jiang Y, Chen Y, Fan L, et al. FGFR1 SUMOylation coordinates endothelial angiogenic signaling in angiogenesis. Proc Natl Acad Sci USA. 2022;119:e2202631119.
5. Dai X, Zhang T, Hua D. Ubiquitination and SUMOylation: protein homeostasis control over cancer. Epigenomics. 2022; 14: 43-58.
6. Shangguan X, He J, Ma Z, Zhang W, Ji Y, Shen K, et al. SUMOylation controls the binding of hexokinase 2 to mitochondria and protects against prostate cancer tumorigenesis. Nat Commun. 2021; 12: 1812.
7. Liang Z, Yang Y, He Y, Yang P, Wang X, He G, et al. SUMOylation of IQGAP1 promotes the development of colorectal cancer. Cancer Lett. 2017; 411: 90-99.
8. Rao HB, Qiao H, Bhatt SK, Bailey LR, Tran HD, Bourne SL, et al. A SUMO-ubiquitin relay recruits proteasomes to chromosome axes to regulate meiotic recombination. Science. 2017; 355: 403-407.
9. Zhou L, Zheng L, Hu K, Wang X, Zhang R, Zou Y, et al. SUMOylation stabilizes hSSB1 and enhances the recruitment of NBS1 to DNA damage sites. Signal Transduc. Target Ther. 2020; 5: 80.
10. Yang Y, Xia Z, Wang X, Zhao X, Sheng Z, Ye Y, et al. Small-molecule inhibitors targeting protein SUMOylation as novel anticancer compounds. Mol Pharmacol. 2018; 9: 885-894.
11. Yewale C, Baradia D, Vhora I, Patil S, Misra A. Epidermal growth factor receptor targeting in cancer: a review of trends and strategies. Biomaterials. 2013; 34: 8690-707.
12. Habban Akhter M, Sateesh Madhav N, Ahmad J. Epidermal growth factor receptor based active targeting: a paradigm shift towards advance tumor therapy. Artif Cells Nanomed Biotechnol. 2018; 46: 1188-1198.
13. Singh D, Attri BK, Gill RK, Bariwal J. Review on EGFR inhibitors: critical updates. Mini Rev Med Chem. 2016; 16: 1134-66.
14. Hoque M, Elmaghrabi YA, Köse, M, Beevi SS, Jose J, Meneses-Salas E, et al. Annexin A6 improves anti-migratory and anti-invasive properties of tyrosine kinase inhibitors in EGFR overexpressing human squamous epithelial cells. FEBS J. 2020; 287: 2961-2978.
15. Pan Q, Lu Y, Xie L, Wu D, Liu R, Gao W, et al. Recent advances in boosting EGFR tyrosine kinase inhibitors-based cancer therapy. Mol Pharm. 2023; 20: 829-852.
16. Boch T, Köhler J, Janning M, Loges S. Targeting the EGF receptor family in non-small cell lung cancer-increased complexity and future perspectives. Cancer Biol Med. 2022; 19: 1543-64.
17. Eno MS, Brubaker JD, Campbell JE, De Savi C, Guzi TJ, Williams BD, et al. Discovery of BLU-945, a reversible, potent, and wild-type-sparing next-generation EGFR mutant inhibitor for treatment-resistant non-small-cell lung cancer. J Med Chem. 2022; 65: 9662-9677.
18. Li D, Wang J, Liu C, Luo Y, Xu H, Wang Y, et al. Making the best use of available weapons for the inevitable rivalry-resistance to EGFR-TKIs. Biomedicines. 2023; 11: 1141.
19. Yamada T, Takeuchi S, Fujita N, Nakamura A, Wang W, Li Q, et al. Akt kinase-interacting protein1, a novel therapeutic target for lung cancer with EGFR-activating and gatekeeper mutations. Oncogene. 2013; 32: 4427-35.
20. Cui Y, Zhu T, Song X, Liu J, Liu S, Zhao R. Downregulation of caveolin-1 increased EGFR-TKIs sensitivity in lung adenocarcinoma cell line with EGFR mutation. Biochem Biophys Res Commun. 2018; 495: 733-739
21. Grewal T, Hoque M, Conway JRW, Reverter M, Wahba M, Beevi SS, et al. Annexin A6-a multifunctional scaffold in cell motility. Cell Adh Migr. 2017; 11: 288-304.
22. Keklikoglou I, Cianciaruso C, Güç E, Squadrito ML, Spring LM, Tazzyman S, et al. Chemotherapy elicits pro-metastatic extracellular vesicles in breast cancer models. Nat Cell Biol. 2019; 21:190-202.
23. Koese M, Rentero C, Kota BP, Hoque M, Cairns R, Wood P, et al. Annexin A6 is a scaffold for PKCα to promote EGFR inactivation. Oncogene. 2013; 32: 2858-72.
24. Vilá de Muga S, Timpson P, Cubells L, Evans R, Hayes TE, Rentero C, et al. Annexin A6 inhibits Ras signalling in breast cancer cells. Oncogene.2009; 28: 363-77.
25. Francia G, Mitchell SD, Moss SE, Hanby AM, Marshall JF, Hart IR. Identification by differential display of annexin-VI, a gene differentially expressed during melanoma progression. Cancer Res. 1996; 56: 3855-8.
26. Grewal T, Evans R, Rentero C, Tebar F, Cubells L, de Diego I, et al. Annexin A6 stimulates the membrane recruitment of p120GAP to modulate Ras and Raf-1 activity. Oncogene. 2005; 24: 5809-20.
27. Li T, Tao Z, Zhu Y, Liu X, Wang L, Du Y, et al. Exosomal annexin A6 induces gemcitabine resistance by inhibiting ubiquitination and degradation of EGFR in triple-negative breast cancer. Cell Death Dis. 2021; 12: 684
28. Xin W, Rhodes DR, Ingold C, Chinnaiyan AM, Rubin MA. Dysregulation of the annexin family protein family is associated with prostate cancer progression. Am J Pathol. 2003;162: 255-61.
29. Sakwe AM, Koumangoye R, Guillory B, Ochieng J. Annexin A6 contributes to the invasiveness of breast carcinoma cells by influencing the organization and localization of functional focal adhesions. Exp Cell Res. 2011; 317: 823-37.
30. Koumangoye RB, Nangami GN, Thompson PD, Agboto VK, Ochieng J, Sakwe AM. Reduced annexin A6 expression promotes the degradation of activated epidermal growth factor receptor and sensitizes invasive breast cancer cells to EGFR-targeted tyrosine kinase inhibitors. Mol. Cancer. 2013; 12: 167.
31. Chen JS, Coustan-Smith E, Suzuki T, Neale GA, Mihara K, Pui CH, et al. Identification of novel markers for monitoring minimal residual disease in acute lymphoblastic leukemia. Blood. 2001; 97: 2115-20.
32. Luo M, Huang Z, Yang X, Chen Y, Jiang J, Zhang L, et al. PHLDB2 mediates cetuximab resistance via interacting with EGFR in latent metastasis of colorectal cancer. Cell Mol Gastroenterol Hepatol. 2022; 13: 1223-1242.
33. Yang Y, Liang Z, Xia Z, Wang X, Ma Y, Sheng Z, et al. SAE1 promotes human glioma progression through activating AKT SUMOylation-mediated signaling pathways. Cell Commun Signa. 2019; 17: 82.
34. Tammsalu T, Matic I, Jaffray EG, Ibrahim AF, Tatham MH, Hay RT. Proteome-wide identification of SUMO modification sites by mass spectrometry. Nat Protoc. 2015;10:1374-88.
35. Yu Y, Sikorski P, Bowman-Gholston C, Cacciabeve N, Nelson KE, Pieper R. Diagnosing inflammation and infection in the urinary system via proteomics. J Transl Med. 2015; 13: 111.
36. Wang X, He Y, Ye Y, Zhao X, Deng S, He G, et al. SILAC-based quantitative MS approach for real-time recording protein-mediated cell-cell interactions. Sci Rep. 2018; 8: 8441.
37. He Y, Wang X, Lu W, Zhang D, Huang L, Luo Y, et al. PGK1 contributes to tumorigenesis and sorafenib resistance of renal clear cell carcinoma via activating CXCR4/ERK signaling pathway and accelerating glycolysis. Cell Death Dis. 2022; 13: 118.
38. Zhang Z, Gao W, Zhou L, Chen Y, Qin S, Zhang L, et al. Repurposing brigatinib for the treatment of colorectal cancer based on inhibition of ER-phagy. Theranostics. 2019; 9: 4878-4892.
39. Chakraborty P, Ahil SB, Jamma T, Yogeeswari P. Combining structure-based and 3D QSAR pharmacophore models to discover diverse ligands against EGFR in oral cancer. Future Med Chem. 2022; 14: 463-478.
40. Todsaporn D, Mahalapbutr P, Poo-Arporn RP, Choowongkomon K, Rungrotmongkol T. Structural dynamics and kinase inhibitory activity of three generations of tyrosine kinase inhibitors against wild-type, L858R/T790M, and L858R/T790M/C797S forms of EGFR. Comput Biol Med. 2022; 147: 105787.
41. García-Melero A, Reverter M, Hoque M, Meneses-Salas E, Koese M, Conway JR, et al. Annexin A6 and late endosomal cholesterol modulate integrin recycling and cell migration. J Biol Chem. 2020; 291: 1320-35.
42. Qi H, Liu S, Guo C, Wang J, Greenaway FT, Sun MZ. Role of annexin A6 in cancer. Oncol Lett. 2015; 10;1947-1952.
43. O'Sullivan D, Dowling P, Joyce H, McAuley E, McCann A, Henry M, et al. A novel inhibitory anti-invasive MAb isolated using phenotypic screening highlights AnxA6 as a functionally relevant target protein in pancreatic cancer. Br J Cancer. 2017; 117: 1326-1335.
44. Sheng Z, Wang X, Ma Y, Zhang D, Yang Y, Zhang P, et al. MS-based strategies for identification of protein SUMOylation modification. Electrophoresis. 2018; 40: 2877-2887.
45. Bao J, Qin M, Mahaman YAR, Zhang B, Huang F, Zeng K, et al. BACE1 SUMOylation increases its stability and escalates the protease activity in Alzheimer's disease. Proc Natl Acad Sci USA. 2018; 115: 3954-3959.
46. Mei L, Yuan L, Shi W, Fan S, Tang C, Fan X, et al.SUMOylation of large tumor suppressor 1 at Lys751 attenuates its kinase activity and tumor-suppressor functions. Cancer Lett. 2017; 386: 1-11.
47. Qu Y, Chen Q, Lai X, Zhu C, Chen C, Zhao X, et al. SUMOylation of Grb2 enhances the ERK activity by increasing its binding with Sos. Mol Cancer. 2014; 13: 95.
48. Fu J, Yu J, Zhang X, Chang Y, Fan H, Dong M, et al. Design, synthesis, and biological evaluation of pyrido[2,3-d]pyrimidine and thieno[2,3-d]pyrimidine derivatives as novel EGFR^L858R/T790M^ inhibitors. J Enzyme Inhib Med Chem. 2023; 38: 2205605.
49. Ullas B, Shrinidhi N, Mansi S, Narayan S, Parveen J, Surender D, et al. All EGFR mutations are (not) created equal: focus on uncommon EGFR mutations. J Cancer Res Clin Oncol. 2023; 149: 1541-1549.
50. Popat S, Hsia TC, Hung JY, Jung HA, Shih JY, Park CK, et al. Tyrosine kinase inhibitor activity in patients with NSCLC harboring uncommon EGFR mutations: a retrospective international cohort study (UpSwinG). Oncologist. 2022; 27: 255-265.
51. Huo D, Wang H, Qin Z, Tian Y, Yan A. Building 2D classification models and 3D CoMSIA models on small-molecule inhibitors of both wild-type and T790M/L858R double-mutant EGFR. Mol Divers. 2022; 26: 1715-1730.
52. Duggirala KB, Lee Y, Lee K. Chronicles of EGFR Tyrosine Kinase Inhibitors: Targeting EGFR C797S Containing Triple Mutations. Biomol Ther (Seoul). 2022; 30:19-27.
53. Liu Q, Yu S, Zhao W, Qin S, Chu Q, Wu K. EGFR-TKIs resistance via EGFR-independent signaling pathways. Mol Cancer. 2018; 17: 53.
54. Yu JJ, Zhou DD, Yang XX, Cui B, Tan FW, Wang J, et al. TRIB3-EGFR interaction promotes lung cancer progression and defines a therapeutic target. Nat Commun. 2020;11:3660.

**Figure legends:**

**Figure 1. The SUMOylated AnxA6 is detected under condition of protein overexpression.** (A-B) The SUMOylated AnxA6 is mainly modified by SUMO1 in HEK293T cells. HEK293T cells were transiently co-transfected with pFlag-AnxA6, pHA-UBC9 and pHis-SUMO1(SUMO2/SUMO3) plasmids as indicated. Cells were collected at 48 h after transfection, and target proteins were captured by IP on anti-Flag antibody coupled agarose beads (A) or Ni^2+^-NTA agarose beads (B). Then the target protein was detected by immunoblotting with anti-Flag and anti-His antibodies. (C-D) The SUMOylation of AnxA6 was detected in response with UBC9/SUMO1 enhancement or inhibitor treatment (2-D08 or ML792). HEK293T cells were co-transiently transfected with pFlag-AnxA6 and pHA-UBC9 or pHis-SUMO1 plasmids as indicated. After transfection for 24h, cells were treated with 150 μM 2-D08 (C) or 10μM ML792 (D) for another 24h. The Flag-tagging AnxA6 protein was enriched by IP on anti-Flag antibody coupled agarose beads (C) or Ni^2+^-NTA agarose beads (D), from which the SUMOylated AnxA6 level was analyzed through western blot using Flag or SUMO1 antibody. S-Flag-AnxA6: SUMOylated Flag-tagging AnxA6, IP: immunoprecipitation, IB: immunoblot, Input: Same account of cell lysate to load. 2-D08 or ML792: SUMOylation inhibitor.

**Figure 2. K299 residue is one main SUMOylation site of AnxA6.** **(A)** The SUMOylated AnxA6 was enriched and detected by His-SUMO1^T95K^ tag. The His-SUMO1^T95K^-AnxA6 conjugated protein was enriched by IP on anti-Flag antibody coupled agarose beads, then the target protein was detected by immunoblotting with anti-Flag and anti-His antibodies. (**B-C**) MS/MS spectra of AnxA6 peptide DAYERDLEADIIGDTSGHFQK^156^, YEK^299^SLYSMIK, NDTSGEYK^314^K in HCD fragmentation mode. And the K156, K299, K314 were identified as the SUMOylation sites. **(D)** Several lysine residues of AnxA6 were predicted to be potential the consensus SUMOylation sites by a bioinformatics software analysis.**(E-G)** Site mutant K299R significantly reduced SUMOylation level of AnxA6. HEK293T cells were co-transfected with plasmids pFlag-AnxA6, each of the single mutants, or pHis-SUMO1 as indicated. After 48 hours later, cell lysates were performed IP to capture Flag-tagging AnxA6 (E) or His-tagging SUMO1 (F), which was immunoblotted with anti-SUMO1 antibody to show SUMOylation level. (G) HEK293T cells were transfected with pFlag-AnxA6 or pFlag-AnxA6^K299R^ plasmids along with pHis-SUMO1 to measure AnxA6 SUMOylation level. The levels of SUMOylation were determined as described in Methods. S-Flag-AnxA6: SUMOylated Flag-tagging AnxA6, IP: immunoprecipitation, IB: immunoblot, Input: same account of cell lysate to load.

**Figure 3. AnxA6 SUMOylation inhibits EGFR/ERK pathway by** **promoting EGFR-PKCα** **interaction. (A-B)** The UBC9 expression enhanced AnxA6-mediated inhibition of pY-EGFR and p-ERK1/2 levels, whereas the inhibitor ML792 treatment abolished suppression of EGFR/ERK phosphorylation due to AnxA6 upon EGF stimulation. (A) HeLa or A431 (B) cells were transfected with pFlag-AnxA6 plasmids for 24h, followed to enhance or inhibit total SUMOylation by pHA-UBC9 plasmid transfection (A) or ML792 inhibitor incubation (B). Cells were serum-starved for overnight and then stimulated with 200 ng/ml (A) or 100 ng/ml EGF for 3 min, then cell lysates were harvested to detect protein expression of pY-EGFR, EGFR, pERK1/2, ERK1/2 and AnxA6.**(C-D)** The K299R mutation of AnxA6 impaired its ability to inactivate pY-EGFR and pERK1/2. HeLa (C) or A431 (D) cells were transfected with pFlag-AnxA6 or pFlag-AnxA6^K299R^ plasmids for 36h, starved overnight and followed with/without 100ng/ml EGF treatment for 3 min. Cell lysates were immunoblotted with the indicated antibodies. **(E)** The K299R mutation impaired its recruitment ability of AnxA6 to promote EGFR-PKCα interaction. A431 cells were transfected with pFlag-AnxA6 or pFlag-AnxA6^K299R^ plasmids for 36h, starved overnight and followed with 100ng/ml EGF treatment for 3 min. One portion of cell lysates was performed IP to capture EGFR-binding complex, detected by western blot analysis with Flag, EGFR and PKCα antibodies. Another part of cell lysates was detected cellular protein level of Flag-AnxA6, EGFR and PKCα. **(F)** HeLa cells were co-transfected with the indicated plasmids for 24h, followed ML792 treatment for 12 h, and cells were subjected to serum deprivation for overnight to stimulate with 100 ng/ml EGF for 3 min. One portion of cell lysates was performed IP to capture Flag-tagging AnxA6 binding protein complex, and detected protein expression by western blot analysis with Flag, SUMO1, EGFR and PKCα antibodies. Another part of cell lysates was detected cellular level of Flag-AnxA6, UbC9, SUMO1, EGFR and PKCα. **(G)** The K299R mutation of AnxA6 impaired its ability to interact EGFR but not affect its binding to PKCα. A431 cells were transfected with pFlag-AnxA6 or pFlag-AnxA6^K299R^ plasmids for 36h, starved overnight and followed with 100ng/ml EGF treatment for 3 min. Cell lysates were immunoprecipitated to capture Flag-tagging AnxA6 and subsequently immunoblotted with indicated antibodies. S-Flag-AnxA6: SUMOylated Flag-tagging AnxA6, IP: immunoprecipitation, IB: immunoblot, Input: same account of cell lysate to load.

**Figure 4. AnxA6 SUMOylation inhibits activation of EGFR mutation-induced signal transduction. (A)** The the silencing of AnxA6 activate pY-GFP-EGFR^T790M/L858R^ and pERK1/2. HeLa stable cells were generated by using lentiviral infections with indicated plasmids, and cells were transfected with pGFP-EGFR^T790M/L858R^ plasmids for 24h, starved overnight and followed with/without 10ng/ml EGF treatment for 3 min. Cell lysates were immunoblotted with the indicated antibodies. **(B)** The K299R mutation of AnxA6 impaired its ability to inactivate pY-EGFR^T790M/L858R^ and pERK1/2. A431 cells were co-transfected with the indicated plasmids for 24h, starved overnight and followed with/without 10ng/ml EGF treatment for 3 min. Cell lysates were immunoblotted with the indicated antibodies. **(C)** The K299R mutation impaired its recruitment ability of AnxA6 to promote GFP-EGFR^T790M/L858R^-PKCα interaction. A431 cells were co-transfected with the indicated plasmids for 24h, starved overnight and followed with/without 10ng/ml EGF treatment for 3 min. Cell lysates were IP to capture GFP-tagging EGFR^T790M/L858R^ and subsequently immunoblotted with indicated antibodies.**(D)**The K299R mutation of AnxA6 impaired its ability to interact GFP-EGFR^T790M/L858R^. A431 cells were co-transfected with the indicated plasmids for 24h, starved overnight and followed with/without 10ng/ml EGF treatment for 3 min. Cell lysates were IP to capture Flag-tagging AnxA6 and subsequently immunoblotted with indicated antibodies. IP: immunoprecipitation, IB: immunoblot, Input: same account of cell lysate to load.

**Figure 5. AnxA6 SUMOylation impedes cell proliferation and migration in EGFR-overexpressing cells by inactivating EGFR-ERK phosphorylation. (A)** The the silencing of AnxA6 weakened the binding of PKCα with EGFR, thereby promoting the EGFR-ERK phosphorylation in HeLa cells. AnxA6-knockdown cell model in HeLa were generated by infecting with lentiviral expressing shRNA targeting to AnxA6, and cells were subjected to serum deprivation for overnight and followed with 100 ng/ml EGF treatment for 3 min. Cell lysates were IP to capture EGFR and subsequently immunoblotted with indicated antibodies. **(B-D)** AnxA6 knockdown promoted HeLa cell proliferation and migration.Cell proliferation **(B-C)** and migration **(D)** were respectively measured on HeLa cells and HeLa-AnxA6(KD) cells. Cells were grown in DEME media containing 0.1% (v/v) FBS and 100 ng/ml EGF. (C) Representative colony formation images (left) and quantification of colonies (right) that compared with HeLa. (D) Representative wound healing images (left) and the calculated cell migration distances (right). **(E)**The K299R mutation of AnxA6 impaired AnxA6 SUMOylation level, which weakened the binding of AnxA6 with EGFR and promoted the EGFR-ERK phosphorylation in A431 cells. A431 stable cells were generated by using lentiviral infections with indicated plasmids, and cells were subjected to serum deprivation for overnight and followed with 100 ng/ml EGF treatment for 3 min. Cell lysates were IP to capture Flag-tagging AnxA6 and subsequently immunoblotted with indicated antibodies. **(F-H)** The K299 mutation of AnxA6 impaired its suppression [activity](../../../../C:/Users/95172/AppData/Local/youdao/dict/Application/8.5.1.0/resultui/html/index.html#/javascript:;) on A431 cell proliferation and migration. Cell proliferation **(F-G)** and cell migration **(H)** were respectively measured on A431 cells, A431-AnxA6 cells and A431-AnxA6^K299R^ cells. Cells were grown in RPMI-1640 media containing 0.1% (v/v) FBS and 100 ng/ml EGF. (G) Representative colony formation images (left) and quantification of colonies (right) that compared with A431. (H) Representative wound healing images (left) and the calculated cell migration distances (right). **(I)** The K299R mutation of AnxA6 impaired its [activity](../../../../C:/Users/95172/AppData/Local/youdao/dict/Application/8.5.1.0/resultui/html/index.html#/javascript:;) in suppressing EGF-induced cyclin D1 expression. Cells (A431, A431-AnxA6, and A431-AnxA6^K299R^) were starved overnight and then treated with 100ng/ml EGF for 0–6h as indicated. Ordinate value, relative cyclin D1 expression (fold of t min/0 min) means the ratio of cyclin D1 between t min and 0 min. Data were represented as the mean ± SD of three separate experiments. *p < 0.05;**p < 0.01; ***p < 0.001. S-Flag-AnxA6: SUMOylated Flag-tagging AnxA6, IP: immunoprecipitation, IB: immunoblot, Input: same account of cell lysate to load.

**Figure 6. AnxA6 SUMOylation promotes** **gefitinib-mediated inhibition of epithelial cancer cell growth and migration.** **(A-C)** AnxA6 knockdown impaired its ability of enhancing anti-proliferation and anti-migratory properties of gefitinib in HeLa cells. The CCK8 (A) and colony-forming (B) assays were used to measure cell proliferation ability of gefitinib-treated HeLa cells. Cells were grown in DMEM media containing 0.1% (v/v) FBS and 100 ng/ml EGF. Quantification of colonies was shown at right, which was calculated as the proportion of cell clone number relative to HeLa. (C) Cell motility was determined by wound healing assay in gefitinib-treated HeLa cells. The bar graphs showed cell migration distances, which were calculated relative to the initial distance before migration (right). **(D-F)**The K299 mutation of AnxA6 impaired its ability of enhancing anti-proliferation and anti-migratory properties of gefitinib in A431 cells. The CCK8 (D) and colony-forming (E) assays were used to measure cell proliferation ability of gefitinib-treated A431 cells. Cells were grown in RPMI-1640 media containing 0.1% (v/v) FBS and 100 ng/ml EGF. Quantification of colonies was shown at right, which was calculated as the proportion of cell clone number relative to A431. (F) Cell motility was determined by wound healing assay in gefitinib-treated A431 cells. The bar graphs showed cell migration distances, which were calculated relative to the initial distance before migration (right). **(G)** The K299 mutation of AnxA6 impaired its ability of enhancing gefitinib-mediated inhibition of EGFR-ERK signaling. The A431, A431-AnxA6, and A431-AnxA6^K299R^ cells were treated with 10μM gefitinib for 24h, and followed with 100ng/ml EGF treatment for 3 min. The levels of pY-EGFR, EGFR, pERK1/2, ERK1/2 and AnxA6 were determined by western blotting. Data were represented as the mean ± SD of three separate experiments. ns, no statistical; *p < 0.05;**p < 0.01; ***p < 0.001.

**Figure 7. AnxA6 SUMOylation inhibits tumor growth of the** **epithelial cancer cells-xenografted nude mouse model.** **(A-D)** AnxA6 knockdown promotes tumor growth in HeLa-xenografted mice.**(A-C)** 5×10^6^ cells, including HeLa and HeLa-AnxA6(KD), were injected subcutaneously into male BALB/c nude mice (n = 5) individually (A**)**. The sizes of tumors were measured at day 6, 9, 12, 15, 18 and 21 after injection (B**)**, and the tumors were weighed (C**)**. And the expression of pY-EGFR, EGFR, pERK1/2, ERK1/2 and AnxA6 were analyzed in HeLa and HeLa-AnxA6(KD)-xenografted tumors. **(E-G)** The K299 mutation of AnxA6 impaired its cancer suppression [effection](../../../../C:/Users/95172/AppData/Local/youdao/dict/Application/8.5.1.0/resultui/html/index.html#/javascript:;) on A431-xenograft tumor growth. 5×10^6^ cells, including A431, A431-AnxA6, and A431-AnxA6^K299R^, were injected subcutaneously into male BALB/c nude mice (n = 5) individually (E**)**. The sizes of tumors were measured at day 9, 12, 15, 18, 21, 24, 27 and 30 after injection (F**)**, and the tumors were weighed (G**)**. **(H)** Ki-67 expression was detected by immunohistochemical staining in A431-xenograft tumors at 30 days after implantation (50×) (left), and the rates of staining positivity for Ki-67 were shown at right. **(I)** The K299R mutation of AnxA6 impaired its ability to inactivate the pY-EGFR and pERK1/2 in A431-xenograft tumors. The expression of pY-EGFR, EGFR, pERK1/2, ERK1/2 and AnxA6 were analyzed in A431, A431-AnxA6, and A431-AnxA6^K299R^-xenografted tumors.T1-T5: tumor tissues from HeLa-xenograft nude mouse.T1-T3: tumor tissues from A431-xenograft nude mouse. *p < 0.05;**p < 0.01.

**Figure 8. A working mechanism model of SUMOylation AnxA6 in EGFR-expressing epithelial cancer cells.** Comparison with the AnxA6 (left) in the un-stimulated cells, the SUMOylated AnxA6 with K299 SUMO1 conjugation (right) is prone to bind EGFR in response to EGF, which facilitates EGFR-PKCα complex formation to decrease the EGF-induced phosphorylation of EGFR-ERK1/2 and cyclin D1 expression, and finally contributes to the stronger inhibition of cell proliferation, migration and tumor growth in the epithelial cancer cells-xenografted nude mouse model, as well as improves gefitinib drug sensitivity.,，.
